# Spatial Transcriptomic Atlas Reveals That Forkhead Box O3‐Mediated Mitochondrial Dynamics Imbalance Drives Premature Ovarian Insufficiency in Mice

**DOI:** 10.1111/acel.70623

**Published:** 2026-07-11

**Authors:** Ziwei Song, Yaoli Yin, Meilin Chen, Xiaolu Jin, Zemin Li, Hongxiao Li, Meihong Shen

**Affiliations:** ^1^ College of Acupuncture Moxibustion and Tuina, College of Health Preservation and Rehabilitation Nanjing University of Chinese Medicine Nanjing China; ^2^ Key Laboratory of Acupuncture and Medicine Research of Ministry of Education Nanjing University of Chinese Medicine Nanjing China

**Keywords:** FOXO3, granulosa cells, mitochondria, premature ovarian insufficiency, spatial transcriptomics

## Abstract

Premature ovarian insufficiency (POI) is a major driver of female reproductive aging, but its mechanisms and the spatial and structural patterns of reproductive aging remain poorly understood. This study, therefore, constructed a spatial transcriptomic atlas of POI mouse models to define the spatial and molecular features of granulosa senescence during disease progression. Spatial analysis revealed disrupted follicular structure and distinct granulosa subpopulations exhibiting blocked differentiation and senescence‐associated gene signatures. Integrating multiple gene sets identified structural and functional mitochondrial impairment, excess fission, reduced fusion, mitochondrial membrane potential loss, insufficient ATP production, and reactive oxygen species accumulation as central features of granulosa senescence in POI. KEGG pathway enrichment implicated FOXO signaling in regulating mitochondrial dysfunction, and FOXO3 phosphorylation was significantly reduced in POI. In a triptolide‐induced KGN cell POI model, pharmacological inhibition of aberrant FOXO3 activation partially restored mitochondrial morphology and function, whereas suppressing FOXO3 phosphorylation in normal KGN cells induced mitochondrial dysfunction. AAV‐mediated FOXO3 overexpression in mouse granulosa cells recapitulated the senescent phenotype and mitochondrial dynamic imbalance, activating PINK1/PARKIN‐mediated mitophagy signaling. Physiologically aged 10‐month‐old mouse ovaries showed identical hallmarks—reduced p‐FOXO3, upregulated senescence markers, and disrupted mitochondrial dynamics—suggesting a conserved feature of ovarian functional decline. Together, these findings demonstrate that aberrant FOXO3 pathway activation disrupts mitochondrial dynamic homeostasis, driving granulosa senescence and ovarian failure in POI. By integrating spatial transcriptomics with functional and mechanistic analyzes, this study establishes a spatially resolved framework for understanding ovarian aging and identifies FOXO3‐regulated mitochondrial pathways as potential diagnostic and therapeutic targets for POI.

## Graphical abstract

This study constructed a spatial transcriptomic atlas of POI mouse ovaries and confirmed that decreased FOXO3 phosphorylation triggers mitochondrial dynamic disorder, granulosa cell senescence and ovarian dysfunction. FOXO3‐mitochondrial axis is a shared core mechanism in both drug‐induced POI and physiological ovarian aging, offering promising therapeutic targets for ovarian aging‐related disorders.

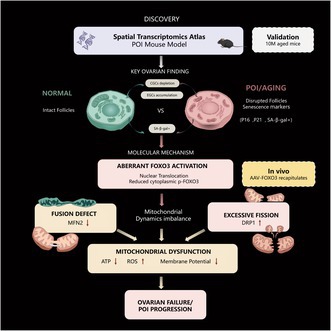

## 1 Introduction

Premature ovarian insufficiency (POI) refers to a decline in ovarian function in women before the age of 40 and is clinically characterized by menstrual abnormalities, elevated follicle‐stimulating hormone levels, and reduced or fluctuating estrogen levels (Hamoda and Sharma 2024). The global prevalence of POI is approximately 3.5% and is increasing, with a shift toward younger age groups (Li et al. 2023). The etiology of POI is complex and includes genetic, autoimmune, iatrogenic, and environmental factors (Guo et al. 2025); however, in many patients the pathogenic mechanisms remain incompletely understood. In addition to impaired reproductive function, POI is associated with long‐term health risks such as osteoporosis, cardiovascular diseases, and cognitive decline, highlighting the need to clarify its underlying mechanisms (Ke et al. 2023).

Granulosa cells (GCs) play essential roles in oocyte development, steroid hormone production, and maintenance of follicular integrity. GC dysfunction promotes follicular atresia, reduces oocyte maturation, and ultimately contributes to ovarian dysfunction in POI (Hu, Wang, et al. 2025). Increasing evidence indicates that cellular senescence of GCs is involved in ovarian dysfunction (Daugelaite et al. 2025); however, the upstream signals that trigger granulosa‐cell senescence during POI progression remain unclear.

Mitochondrial dysfunction is a key hallmark of granulosa‐cell senescence (Adlimoghaddam 2025). It is characterized by altered mitochondrial morphology, reduced energy production, increased reactive oxygen species (ROS) generation, and decreased mitochondrial membrane potential (MMP), all of which induce cellular damage and promote senescence (Zhang et al. 2025). Maintenance of mitochondrial function depends on balanced mitochondrial dynamics, including fission and fusion. Excessive fission leads to mitochondrial fragmentation and accelerates tissue aging (You et al. 2023), whereas enhanced fusion helps restore MMP and reverse aging‐related phenotypes (Wu et al. 2025). Forkhead box O3 (FOXO3), a transcription factor that regulates lifespan and metabolism, is a key upstream regulator of mitochondrial function (Klinpudtan et al. 2022). Under stress, dephosphorylated FOXO3 translocates to the nucleus and regulates downstream genes, including those involved in mitochondrial dynamics (Li et al. 2022). However, whether FOXO3 regulates granulosa‐cell senescence through mitochondrial dynamics during POI development remains unknown.

Distinct regions and cell populations within the ovary perform specialized functions. Conventional transcriptomic approaches cannot simultaneously resolve gene expression and spatial localization, whereas spatial transcriptomics enables direct mapping of transcripts within tissue sections through high‐throughput sequencing (Salmén et al. 2018). This approach links molecular pathways to specific cellular locations and tissue microenvironments, bridging the gap between single‐cell omics and histopathology, and has been widely applied to characterize spatiotemporal patterns in tumors (Marco Salas et al. 2025).

This study aimed to investigate the spatiotemporal changes in GCs and key regulatory genes during POI development. Using a tripterygium glycoside‐induced mouse POI model, we constructed an ovarian spatial atlas and identified senescent GC subtypes. Our results show that FOXO3 induces granulosa cell senescence by regulating mitochondrial dynamics, leading to structural and functional mitochondrial abnormalities, which contribute to POI progression.

## 2 Results

### 2.1 Xenium‐Based Spatial Transcriptomics Deciphers Cellular Atlas and Follicular Spatial Disorganization in Murine POI Ovaries

**FIGURE 1:**
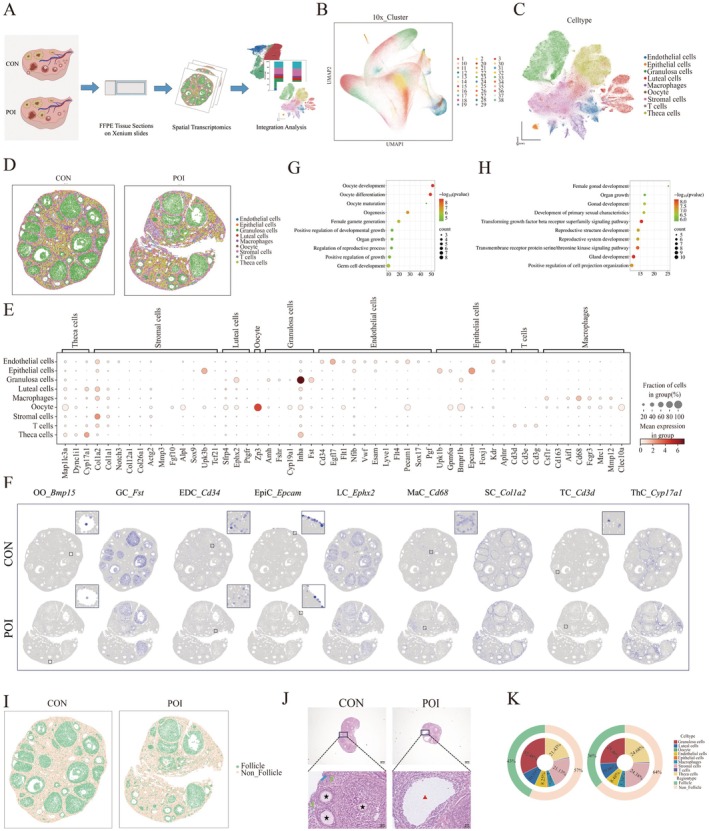
Spatial Transcriptomic Analysis of Mouse Ovaries. (A) Study workflow. (B, C) UMAP plots showing 38 Clusters and 9 cell types. (D) Spatial distribution of cell populations in ovarian tissues: Left panel represents ovaries from the CON group, and right panel represents ovaries from the POI group. (E) Expression levels of representative marker genes of the 9 cell populations across different cell groups in ovarian tissues. (F) Spatial localization of representative marker genes of the 9 cell populations in the CON and POI groups. Each row represents one cell type, and each column represents different spatial regions of cell populations in the samples; colors from light to dark indicate gradually increasing expression levels. OO: Oocytes; GC: Granulosa cell; EDC: Endothelial cell; Epic: Epithelial cell; LC: Luteal cell; Mac: Macrophage; SC: Stromal cell; TC: T cell; Thc: Theca cell. (G) GO enrichment analysis of the top 50 marker genes of OO. (H) GO enrichment analysis of the top 50 marker genes of GCs. (I) Distribution of the follicular region and non‐follicular region in ovaries of the CON and POI groups; the green region represents the follicular region. (J) HE staining images of ovaries from the CON and POI groups: Blue arrows: Primordial follicles; green arrows: Primary follicles; black pentagrams: Secondary follicles; red triangles: Atretic follicles. Scale bar = 200 μm; scale bar in higher‐magnification images = 20 μm. (K) Pie charts showing the proportions of the follicular region, non‐follicular region (outer layer), and cell type composition (inner layer) in ovaries of the CON and POI groups.

Ovaries from blank control (CON) mice and tripterygium glycosides (TG)‐treated mice were subjected to spatial transcriptomic sequencing to map ovarian cell distributions (Figures 1A and S1A). Dimensionality reduction and clustering analyzes identified 38 distinct cell subpopulations (Figure 1B). Based on known marker genes (Table S1), nine major cell types were annotated: oocytes expressing *Bmp15* and *Zp3*, GCs expressing *Fst* and *Amh*, endothelial cells expressing *Cd34*, epithelial cells expressing *Epcam*, luteal cells expressing *Ephx2*, macrophages expressing *Cd68*, stromal cells expressing *Col1a2*, T cells expressing *Cd3d*, and theca cells expressing *Cyp17a1* (Figure 1C). Each population localized to its expected anatomical region, indicating that spatial transcriptomics effectively delineated ovarian spatial organization (Figure 1C–F). Gene Ontology (GO) enrichment analysis of the top 50 marker genes of each cell type showed that oocyte markers were enriched in “oocyte development”‐related pathways, whereas GC markers were enriched in “gonad development,” consistent with their known biological functions (Figures 1G,H and S1B). To assess ovarian spatial architecture in POI, oocytes, GCs, theca cells, and luteal cells were defined as the follicular region, and all remaining cells as the non‐follicular region (Figure 1I). Compared with CON ovaries, POI ovaries exhibited a significant expansion of the non‐follicular region and a reduced number of follicles, in agreement with hematoxylin and eosin (HE) staining (Figure 1J). Statistical comparison also revealed a reduction in GC proportion in POI ovaries (Figure 1K). Together with the increased number of atretic follicles (Figure S1C), these findings indicate a markedly disrupted spatial microenvironment in POI ovaries.

### 2.2 Dysregulated Differentiation Trajectories of GCs Drive a Multilayered Senescence Landscape in POI

**FIGURE 2:**
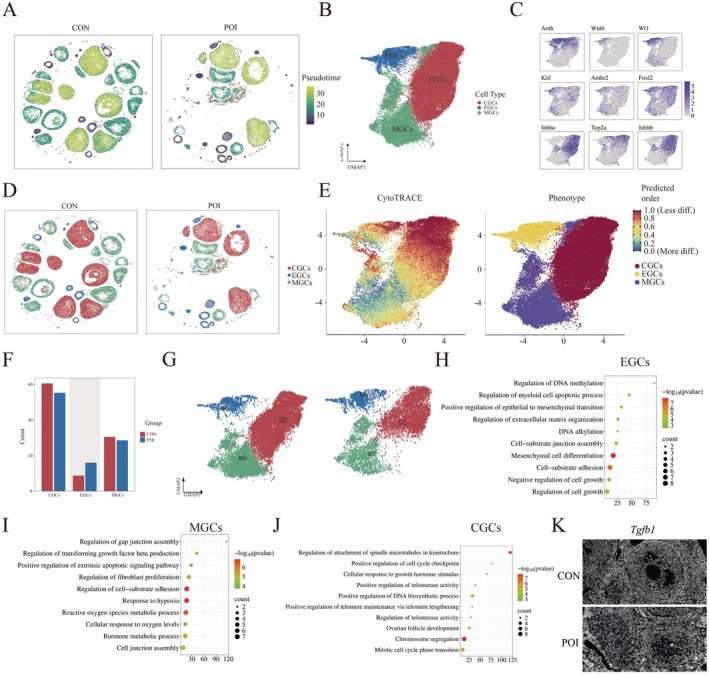
Spatial Pseudotime Analysis and Subtype Identification of Ovarian GCs. (A) Spatial mapping of Monocle3 pseudotime analysis for GCs in CON and POI groups, with different colors representing distinct pseudotemporal developmental stages of GCs; (B) Monocle3 pseudotemporal trajectory analysis of GCs, where different colors distinguish three GC subtypes: EGCs, MGCs, and CGCs; (C) Spatial expression heatmap of subtype‐specific markers for GCs, with rows representing stage‐specific marker genes, columns corresponding to different GC subtypes, and color intensity reflecting gene expression levels, illustrating the enrichment characteristics of each marker within subtypes; (D) Spatial localization of the three GC subtypes in CON and POI ovarian tissues, with different colors representing CGCs (red), EGCs (blue), and MGCs (green); (E) Analysis of differentiation potential Cytotrace and phenotype of GC subtypes, where in the left panel, color intensity represents the Cytotrace stemness score, and in the right panel, different colors distinguish GC subtypes; (F) Bar chart showing the proportional abundance of the three GC subtypes in CON and POI groups; (G) Comparative spatial mapping of GC pseudotemporal trajectories between CON and POI groups; (H) Bubble plot of GO enrichment analysis for differentially expressed genes in EGCs; (I) Bubble plot of GO enrichment analysis for differentially expressed genes in MGCs; (J) Bubble plot of GO enrichment analysis for differentially expressed genes in CGCs; (K) Spatial expression mapping of the pro‐senescence factor *Tgfb1* in CON and POI ovarian tissues, with white indicating nuclei and blue indicating the senescence factor *Tgfb1*. Scale bar =200 μm.

GCs are central regulators of follicular development; however, their senescence trajectory in POI remains poorly defined. Using Xenium spatial transcriptomic data, Monocle3 pseudotime analysis identified three functionally distinct GC types: early GCs (EGCs, *Amh*), mural GCs (MGCs, *Kitl*), and cumulus GCs (CGCs, *Inhba*) (Figures 2A,B and S2A). Stage‐specific markers displayed dynamic expression, with *Wnt6* and *Wt1* enriched in EGCs, *Amhr2* and *Foxl2* in MGCs, and *Top2a* and *Inhbb* in CGCs (Figure 2C). Spatial mapping confirmed their anatomical localization: EGCs predominated in primordial and primary follicles (developmental initiation), MGCs in secondary and early antral follicles (middle developmental stage), and CGCs in mature antral follicles (developmental endpoint) (Figure 2D). CytoTrace analysis validated these differentiation states, with CGCs showing the highest differentiation scores, followed by EGCs and MGCs (Figures 2E and S2B). In POI ovaries, the hierarchical organization of GCs was pathologically remodeled. Pseudotime trajectories revealed accumulation of EGCs at early developmental stages, marked depletion of CGCs, and a reduction in MGCs, resulting in exhaustion of functionally mature GCs (Figure 2F,G). GO enrichment analysis of differentially expressed genes revealed subtype‐specific senescence programs. EGCs were enriched in “regulation of extracellular matrix organization” and “mesenchymal cell differentiation,” consistent with a profibrotic senescence‐associated secretory phenotype (SASP). MGCs were enriched in “reactive oxygen species metabolic process” and “regulation of gap junction assembly,” indicating impaired intercellular communication. CGCs were enriched in “meiotic cell cycle process” and “positive regulation of telomerase activity,” linking them to replicative senescence (Figure 2H–J). Spatial transcriptomic mapping showed increased expression of pro‐senescence genes (*Tgfb1*, *Trp53*, *Rb1*) and decreased expression of protective genes (*Sod2*, *Tert*) within the GC layer of POI ovaries (Figures 2K and S2C). Collectively, these results demonstrate that GC subtypes in POI ovaries display distinct senescence‐associated transcriptional programs in a spatially resolved manner, which may collectively contribute to premature depletion of the ovarian reserve.

### 2.3 Ovarian GC Senescence in POI Is Associated With Structural and Functional Mitochondrial Damage

**FIGURE 3:**
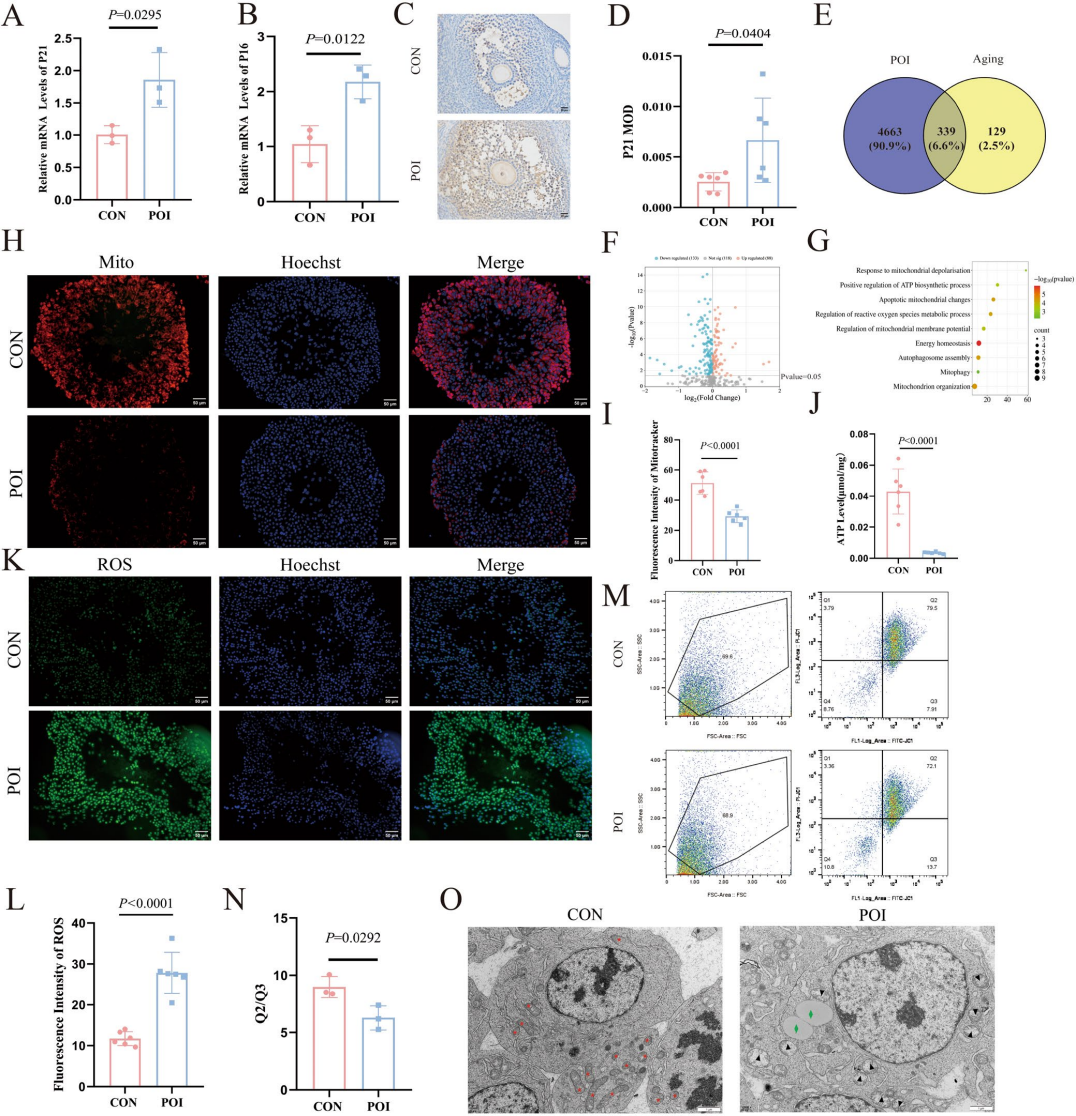
Senescence phenotype and mitochondrial damage in GCs of POI ovaries. (A) *P21* mRNA expression levels in ovarian GCs of the CON and POI groups, normalized to *Gapdh*; *n =* 3 mice per group. (B) *P16* mRNA expression levels in ovarian GCs of the CON and POI groups, normalized to *Gapdh*; *n =* 3 mice per group. (C) Representative immunohistochemical staining of P21 in the ovaries of the CON and POI groups. (D) Quantification of P21 MOD values; *n =* 6 mice per group. (E) Venn diagram showing the overlap between DEGs identified in POI and the mouse senescence gene set from the Aging Atlas database. (F) Volcano plot of DEGs between the POI and CON groups. (G) GO enrichment analysis bubble plot of DEGs in POI. (H) Fluorescence images of mitochondrial activity in GCs stained with MitoTracker Red CMXRos (red); nuclei labeled with Hoechst (blue); scale bar = 50 μm. (I) Quantification of MitoTracker fluorescence intensity; *n =* 6 mice per group. (J) Quantification of ATP levels in GCs; *n =* 6 mice per group. (K) Fluorescence images of ROS in GCs labeled with DCFH‐DA (green); nuclei labeled with Hoechst (blue); scale bar = 50 μm. (L) Quantification of ROS fluorescence intensity; *n =* 6 mice per group. (M) Left: Contour plots with gating strategy of JC‐1 assay for mitochondrial membrane potential; Right: Corresponding flow cytometry scatter plots. (N) Quantification of JC‐1 fluorescence Q2/Q3 ratio; *n =* 3 mice per group. (O) Transmission electron microscopy images of mitochondria in GCs; red asterisks: Normal mitochondria; black arrowheads: Swollen mitochondria; green diamonds: Lipid droplets; scale bar = 1 μm.

To investigate the senescent phenotype of GCs in POI, we first examined the expression of classical senescence markers P16 and P21. qRT‐PCR results showed that *P* *21* and *P* *16* mRNA levels in the POI group were higher than in the CON group (Figure 3A,B). Immunohistochemical staining further confirmed increased P21 expression in POI tissues, with MOD values higher than those in the CON tissues (Figure 3C,D and S4C).

To elucidate the upstream regulatory network underlying this senescent phenotype, we integrated the DEGs identified by spatial transcriptomics with the mouse senescence gene set from the Aging Atlas database, identifying 339 overlapping genes of which 221 reached statistical significance: 88 genes were upregulated, and 133 were downregulated in the POI group (Figure 3E,F). GO enrichment analysis revealed that these DEGs were enriched in biological processes, including response to mitochondrial depolarization, regulation of mitochondrial membrane potential, positive regulation of ATP biosynthetic process, and energy homeostasis, suggesting a close association between GC senescence and mitochondrial structural and functional damage (Figure 3G).

We subsequently assessed mitochondrial structure and function in ovarian GCs of the POI mouse model. MitoTracker Red CMXRos labelling of functional mitochondria showed weaker fluorescence intensity in POI group GCs than in the CON group GCs, indicating impaired mitochondrial activity (Figure 3H,I). Consistently, ATP quantification confirmed reduced ATP levels in POI GCs (Figure 3J). DCFH‐DA staining revealed elevated ROS levels in POI GCs (Figure 3K,L). Flow cytometric assessment of MMP showed that the proportion of red fluorescent cells (Q2) was reduced, while that of green fluorescent cells (Q3) was increased, in POI group GCs compared with CON group GCs, resulting in a lower Q2/Q3 ratio (Figure 3M,N). Transmission electron microscopy further confirmed mitochondrial damage: GCs in the CON group displayed intact mitochondria with well‐defined cristae, whereas those in the POI group exhibited obvious mitochondrial swelling with blurred or absent cristae (Figure 3O).

Taken together, these results demonstrate that GC senescence in POI is accompanied by mitochondrial structural damage and functional impairment.

### 2.4 GCs in POI Ovaries Exhibit Abnormal Mitochondrial Dynamics, Which Sequencing and Database Analyzes Implicate in a Potential Association With FOXO3

**FIGURE 4:**
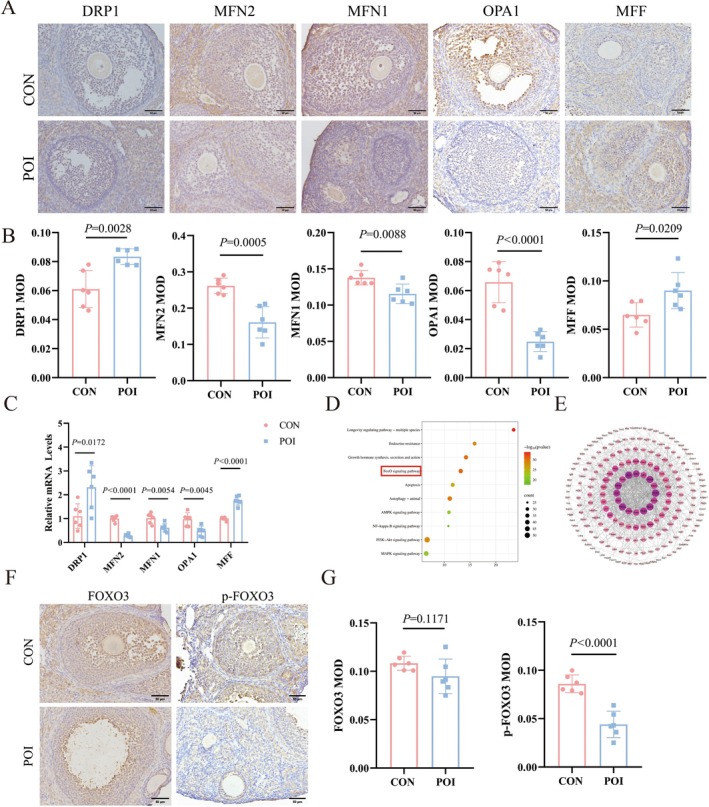
Sequencing and database analyzes suggest that FOXO3 may be involved in mitochondrial dysfunction of GCs in POI. (A) Representative immunohistochemical staining images of mitochondrial dynamics‐related proteins (DRP1, MFN2, MFN1, OPA1, MFF) in ovaries from the CON and POI groups; top row: CON group; bottom row: POI group; scale bar=50μm; (B) Quantitative analysis of the mean optical density (MOD) for mitochondrial dynamics‐related proteins; (C) qRT‐PCR analysis of mRNA expression of mitochondrial dynamics‐related genes, normalized to *Gadph*; *n =* 6 mice per group; (D) Bubble plot of KEGG pathway enrichment analysis for the overlapping genes between POI differentially expressed genes and the mouse aging gene set from the Aging Atlas database; (E) PPI network of the overlapping genes; (F) Representative immunohistochemical staining images of FOXO3 and p‐FOXO3 in ovaries from the CON and POI groups; scale bar=50μm; (G) Quantitative analysis of the MOD for FOXO3 and p‐FOXO3; *n =* 6 mice per group.

Impaired mitochondrial dynamic homeostasis is a key driver of mitochondrial structural and functional defects (Zanfardino et al. 2025; Hu, Liu, et al. 2025). Immunostaining showed that, compared with CON ovaries, POI GCs exhibited increased levels of the fission‐promoting proteins DRP1 and MFF, as indicated by higher average absorbance values, whereas the fusion‐related proteins MFN2, MFN1, and OPA1 showed reduced staining intensity (Figures 4A,B and S4A). Consistently, qRT‐PCR results showed downregulation of *Mfn2*, *Mfn1*, and *Opa1* and upregulation of *Drp1* and *Mff* in POI GCs, consistent with previous reports (Figure 4C).

KEGG pathway enrichment analysis of DEGs associated with mitochondrial dysfunction revealed significant enrichment of the FOXO signaling pathway (Figure 4D). Protein–protein interaction (PPI) network analysis identified *FOXO3*, *AKT1*, and *PIK3R2* as central regulatory genes (Figure 4E). Given the established role of FOXO3 in regulating cellular senescence and metabolism (Cao et al. 2023), we further examined its expression. Immunohistochemical analysis showed no significant difference in total FOXO3 protein levels between the POI and CON GCs, whereas phosphorylated FOXO3 was significantly reduced in the POI group (Figures 4F,G and S4B). These findings suggest a potential link between aberrant FOXO3 activation and mitochondrial dynamic imbalance in POI group ovaries. To further characterize this association in a more controlled cellular environment, we established an in vitro POI model using triptolide‐treated KGN cells.

### 2.5 The Triptolide‐Induced POI Cell Model Recapitulates FOXO3 Activation and Mitochondrial Dynamic Imbalance

**FIGURE 5:**
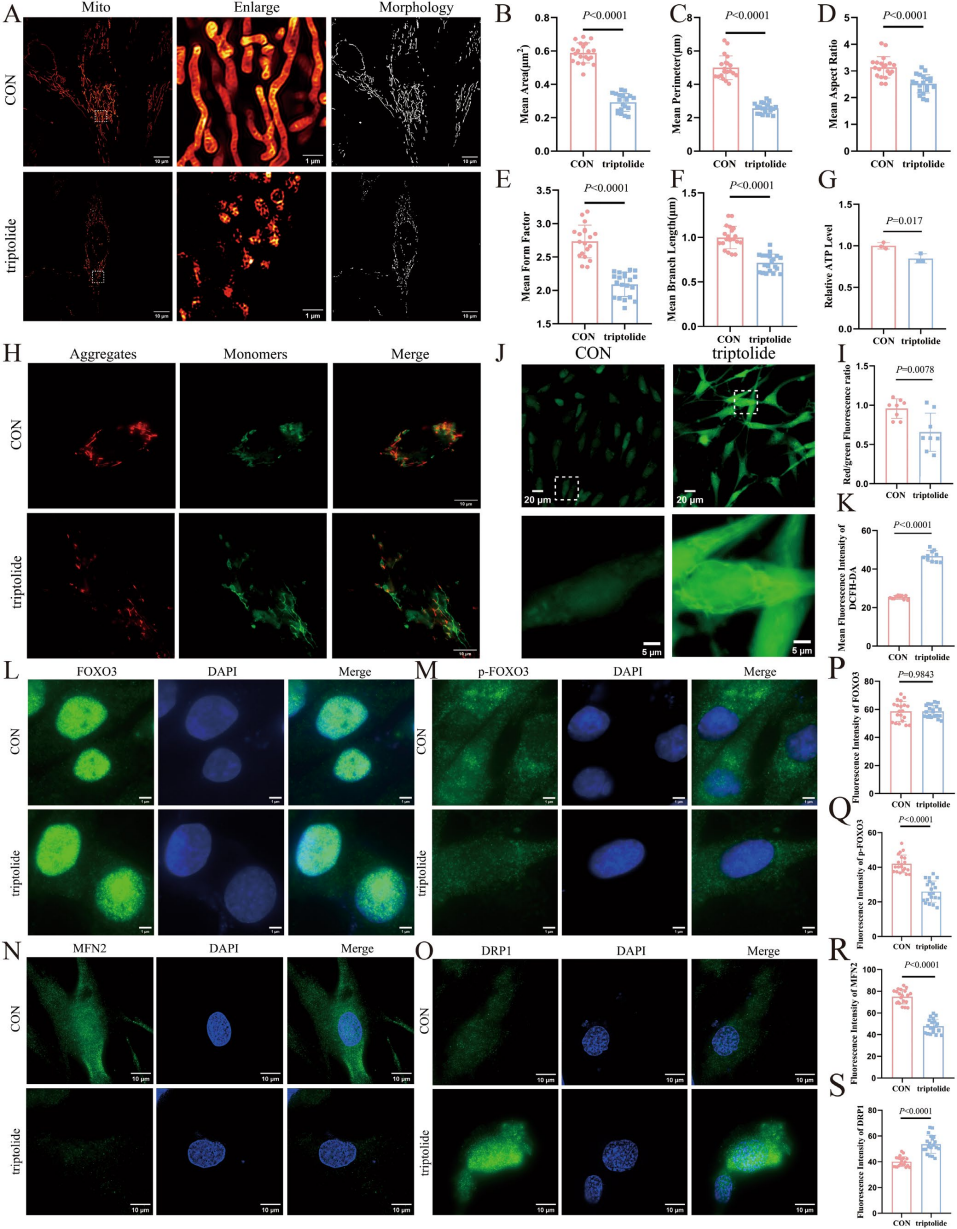
Dysregulation of FOXO3 and Mitochondrial Dysfunction in a Triptolide‐Induced POI Cell Model (A) Mitochondrial morphology in KGN cells stained with PK Mito and observed under Structured Illumination Microscopy; scale bar = 10 μm, inset scale bar = 1 μm; (B–F) Statistical analysis of mitochondrial average area, perimeter, branch length, aspect ratio, and form factor; *n =* 20 biological replicates; (G) Quantitative analysis of ATP levels in KGN cells; *n =* 3 biological replicates; (H) MMP in KGN cells stained with JC‐1 and observed under Structured Illumination Microscopy, aggregates (red) and monomers (green), scale bar = 10 μm; (I) Quantitative analysis of the JC‐1 red/green fluorescence intensity ratio; *n =* 8 biological replicates; (J) ROS detection in KGN cells stained with DCFH‐DA (green) under fluorescence microscopy, scale bar = 20 μm, inset scale bar = 5 μm; (K) Quantitative analysis of ROS fluorescence intensity, fluorescence intensity was quantified from 6 fields of view per biological replicate; *n =* 10 biological replicates; (L–O) Immunofluorescence staining of FOXO3, p‐FOXO3, MFN2, and DRP1 (green) in KGN cells under Structured Illumination Microscopy, nuclei stained with DAPI (blue), figure L, M scale bar = 1 μm, figure N, O scale bar = 10 μm; (P–S) Quantitative analysis of fluorescence intensity for FOXO3, p‐FOXO3, MFN2, and DRP1; *n =* 20 biological replicates.

A POI model was established by treating KGN cells with triptolide (Figure S5A). Super‐resolution microscopy revealed an elongated perinuclear mitochondrial network in the CON group, whereas triptolide‐treated cells displayed fewer networks and punctate, fragmented mitochondria distributed throughout the cytoplasm (Figure 5A). Quantitative analysis of mitochondrial morphology showed that triptolide significantly reduced mitochondrial mean area, perimeter, branch length, aspect ratio, and form factor compared with CON cells, indicating marked structural damage (Figure 5B–F). Mitochondrial functional assays further confirmed impairment. ATP production was significantly reduced in triptolide‐treated cells (Figure 5G). JC‐1 staining revealed decreased red fluorescence, increased green fluorescence, and a significantly reduced red‐to‐green fluorescence ratio, indicating loss of MMP (Figure 5H,I). DCFH‐DA staining showed a significant increase in ROS (Figure 5J,K). Together, these results demonstrate severe mitochondrial dysfunction in triptolide‐treated KGN cells.

Total FOXO3 protein levels did not differ significantly between triptolide‐treated and CON cells (Figure 5L,P), whereas p‐FOXO3 was significantly reduced (Figure 5M,Q), as confirmed by immunofluorescence. Western blotting (Figure S5B,C) revealed that the ratio of p‐FOXO3 to total FOXO3 was reduced in triptolide‐treated cells compared with control cells. In parallel, mitochondrial fusion protein MFN2 showed decreased fluorescence, whereas the fission protein DRP1 was increased (Figure 5N,O,R,S).

Thus, the triptolide‐induced POI cell model reproduces mitochondrial fragmentation, functional failure, and abnormal FOXO3 activation observed in POI tissue. This consistent in vitro platform allows for further investigation into whether FOXO3 directly orchestrates the disruption of mitochondrial fission–fusion homeostasis.

### 2.6 FOXO3 Hyperactivation Is Pathogenic, Whereas Its Inhibition Shows Therapeutic Potential

**FIGURE 6:**
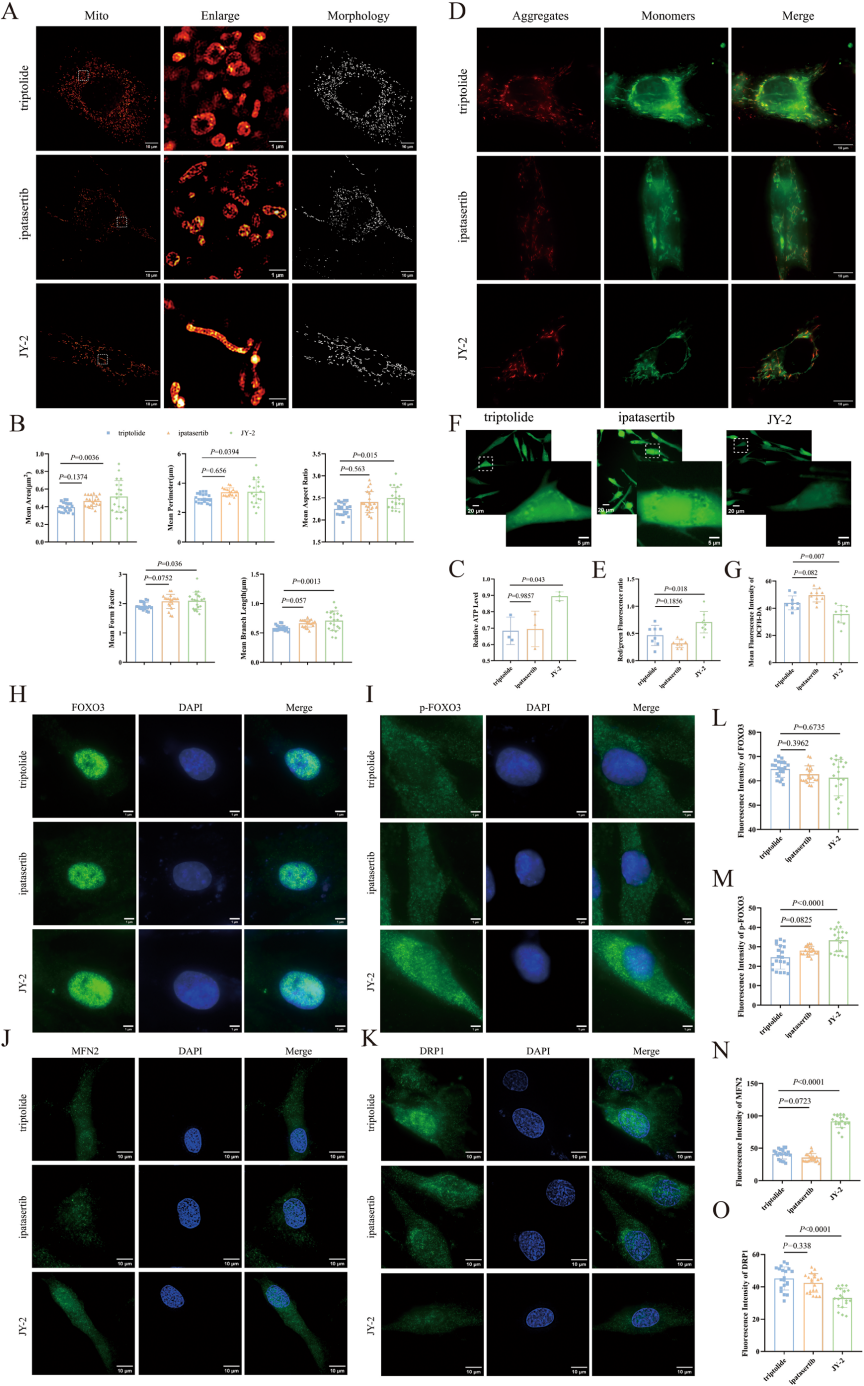
Effect of FOXO3 Functional Regulation on Cellular Mitochondrial Dynamics (A) Mitochondrial morphology in KGN cells stained with PK Mito and observed under Structured Illumination Microscopy, scale bar = 10 μm, inset scale bar = 1 μm; (B) Statistical analysis of mitochondrial average area, perimeter, branch length, aspect ratio, and form factor; *n* = 20 biological replicates; (C) Quantitative analysis of ATP levels in KGN cells; *n* = 3 biological replicates; (D) MMP in KGN cells stained with JC‐1 and observed under Structured Illumination Microscopy, aggregates (red), monomers (green), scale bar = 10 μm; (E) Quantitative analysis of JC‐1 red/green fluorescence intensity ratio; *n* = 8 biological replicates; (F) ROS detection in KGN cells stained with DCFH‐DA (green) under fluorescence microscopy, scale bar = 20 μm, inset scale bar = 5 μm; (G) Quantitative analysis of ROS fluorescence intensity, fluorescence intensity was quantified from 6 fields of view per biological replicate; *n* = 10 biological replicates; (H–K) Immunofluorescence staining of FOXO3, p‐FOXO3, MFN2, and DRP1 (green) in KGN cells under Structured Illumination Microscopy, nuclei stained with DAPI (blue),figure H, I scale bar = 1 μm, figure J, K scale bar = 10 μm; (L–O) Quantitative analysis of fluorescence intensity for FOXO3, p‐FOXO3, MFN2, and DRP1; *n* = 20 biological replicates.

To further define the role of FOXO3 in mitochondrial dysfunction during POI, gain‐ and loss‐of‐function experiments were performed. Normal KGN cells were treated with the AKT inhibitor ipatasertib, which induces FOXO3 hyperactivation by blocking its phosphorylation, whereas triptolide‐induced POI cells were pretreated with the FOXO3 inhibitor JY‐2 (Sun et al. 2018; Choi et al. 2021) (Figure S6A,B). Super‐resolution microscopy showed that ipatasertib treatment in normal cells induced mitochondrial swelling, vacuolization, and fragmentation, closely resembling the triptolide‐induced POI phenotype (Figure 6A). In contrast, JY‐2 partially restored mitochondrial network continuity and reduced fragmented structures. Morphometric analysis confirmed that mitochondrial mean area, perimeter, branch length, aspect ratio, and form factor in normal cells after ipatasertib treatment were comparable to those in the POI cells, whereas JY‐2 significantly improved these parameters in POI cells (Figure 6B). Mitochondrial function assays revealed that normal cells treated with ipatasertib exhibited mitochondrial damage phenotypes consistent with POI cells,as evidenced by altered ATP (Figure 6C), MMP (Figure 6D,E), and ROS levels (Figure 6F,G). Conversely, JY‐2 treatment reversed these functional defects in the POI cells by increasing ATP levels, restoring MMP levels, and reducing ROS levels. The p‐FOXO3 level in ipatasertib‐treated cells was comparable to that in POI cells, whereas JY‐2 markedly upregulated p‐FOXO3 expression in POI cells (Figure 6H,I,L,M), which was consistent with the Western blot quantification results (Figure S6C,D). Consistently, ipatasertib induced mitochondrial dynamic imbalance, while JY‐2 restored the balance between mitochondrial fusion and fission in POI cells (Figure 6J,K,N,O). These results indicate that excessive FOXO3 activation disrupts mitochondrial dynamics and drives POI‐associated mitochondrial dysfunction, whereas FOXO3 inhibition can partially reverse these pathological changes.

### 2.7 In Vivo FOXO3 Overexpression Causally Drives Mitochondrial Dynamic Imbalance and Enhances Mitophagy Signaling

**FIGURE 7:**
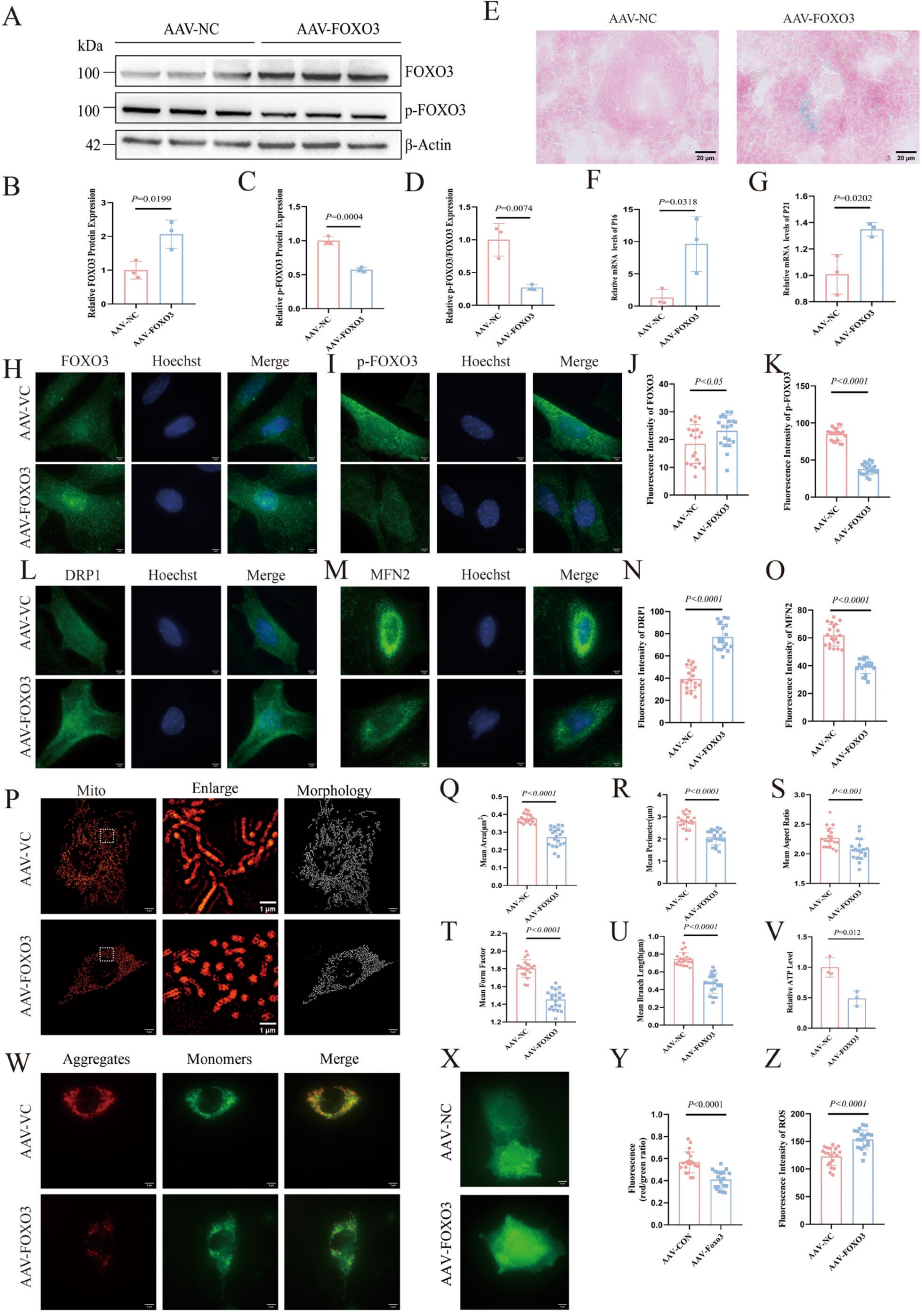
FOXO3 overexpression induces mitochondrial dynamic imbalance and dysfunction. (A) Representative Western blot bands of total FOXO3 and phosphorylated FOXO3 (p‐FOXO3) protein expression in ovarian tissues from the AAV‐NC and AAV‐FOXO3 groups. (B) Quantitative statistical analysis of the relative expression level of total FOXO3 protein, *n =* 3 mice per group. (C) Quantitative statistical analysis of the relative expression level of p‐FOXO3 protein, *n =* 3 mice per group. (D) Quantitative statistical analysis of the ratio of p‐FOXO3 to total FOXO3 protein, *n =* 3 mice per group. (E) Representative SA‐β‐gal staining images of ovarian tissues from the AAV‐NC and AAV‐FOXO3 groups; scale bar = 20 μm. (F) Quantitative detection of the relative mRNA expression level of senescence marker gene *P* *16* in GCs of the two groups. (G) Quantitative detection of the relative mRNA expression level of senescence marker gene *P* *21* in GCs of the two groups. (H) Representative immunofluorescence images of FOXO3 (green) in GCs; cell nuclei were stained with Hoechst (blue); scale bar = 5 μm. (I) Representative immunofluorescence images of p‐FOXO3 (green) in GCs; cell nuclei were stained with Hoechst (blue); scale bar = 5 μm. (J) Quantitative analysis of FOXO3 fluorescence intensity in the AAV‐NC and AAV‐FOXO3 groups, *n =* 20 biological replicates. (K) Quantitative analysis of p‐FOXO3 fluorescence intensity in the AAV‐NC and AAV‐FOXO3 groups, *n =* 20 biological replicates. (L) Representative immunofluorescence images of DRP1 (green) in GCs; cell nuclei were stained with Hoechst (blue); scale bar = 5 μm. (M) Representative immunofluorescence images of MFN2 (green) in GCs; cell nuclei were stained with Hoechst (blue); scale bar = 5 μm. (N) Quantitative analysis of DRP1 fluorescence intensity in the two groups, *n =* 20 biological replicates. (O) Quantitative analysis of MFN2 fluorescence intensity in the two groups, *n =* 20 biological replicates. (P) Super‐resolution structured illumination microscopy images of mitochondria stained by MitoTracker, including original field of view, partial enlarged view and mitochondrial morphological binarization analysis image; scale bar = 5 μm, inset scale bar = 1 μm. (Q–U) Quantitative statistical analysis of mitochondrial mean area, mean perimeter, mean aspect ratio, mean form factor and mean branch length in the two groups, *n =* 20 biological replicates. (V) Quantitative detection of relative ATP levels in GCs of the two groups, *n =* 3 biological replicates. (W) Representative JC‐1 staining images for mitochondrial membrane potential detection, red fluorescence represents JC‐1 aggregates, green fluorescence represents JC‐1 monomers; scale bar = 5 μm. (X) Representative DCFH‐DA immunofluorescence images for intracellular ROS detection (green fluorescence); scale bar = 5 μm. (Y) Quantitative analysis of JC‐1 red/green fluorescence ratio in GCs, *n =* 20 biological replicates. (Z) Quantitative analysis of intracellular ROS fluorescence intensity in GCs, *n =* 20 biological replicates.

To establish a causal link between FOXO3 activation and mitochondrial dynamic imbalance in vivo, AAV‐FOXO3 was delivered via in situ intraovarian injection for FOXO3 overexpression in granulosa cells, with AAV‐NC used as the control. Western blot confirmed that total FOXO3 protein levels in the ovaries of the AAV‐FOXO3 group were elevated compared to the AAV‐NC group, validating successful overexpression (Figure 7A–D). Immunofluorescence staining of dispersed cumulus cells revealed that FOXO3 levels were elevated (Figure 7H,J), while p‐FOXO3 levels were reduced (Figure 7I,K) in the AAV‐FOXO3 group, further confirming the overexpression of the mouse model construction.

To clarify whether in vivo FOXO3 overexpression is sufficient to trigger GC senescence, we further detected canonical senescence markers in the AAV‐FOXO3 mouse model. SA‐β‐gal staining of ovarian sections revealed abundant blue senescence‐positive regions in the AAV‐FOXO3 group (Figure 7E). qRT‐PCR quantification further demonstrated significant upregulation of senescence marker *P16* (Figure 7F) and *P21* (Figure 7G) mRNA in granulosa cells from the AAV‐FOXO3 group versus AAV‐NC. Collectively, these findings verify that AAV‐mediated FOXO3 overexpression induces a senescent phenotype in mouse GCs.

We subsequently observed that FOXO3 overexpression was accompanied by an imbalance in mitochondrial dynamics‐related proteins: DRP1 fluorescence intensity was elevated (Figure 7L,N), while MFN2 fluorescence intensity was reduced (Figure 7M,O) in cumulus cells of the AAV‐FOXO3 group. Consistently, the mRNA levels of these two genes in ovarian GCs showed identical expression trends (Figure 7N,O), indicating excessive mitochondrial fission.

Super‐resolution structured illumination microscopy revealed that the mitochondrial network in GCs of the AAV‐FOXO3 group exhibited more pronounced fragmentation than the elongated mitochondrial network observed in the AAV‐NC group (Figure 7P). Quantitative analysis confirmed that mean mitochondrial area, perimeter, aspect ratio, form factor, and branch length were reduced in the AAV‐FOXO3 group (Figure 7Q–U), accompanied by decreased ATP levels (Figure 7V). JC‐1 staining showed a reduced red/green fluorescence ratio in AAV‐FOXO3 GCs, indicating MMP loss (Figure 7W,Y). DCFH‐DA staining revealed elevated ROS levels in the AAV‐FOXO3 group (Figure 7X,Z).

Mitochondrial dysfunction and dynamic homeostasis disruption frequently trigger mitophagy, a selective process for eliminating damaged mitochondria (Chen et al. 2023). To further clarify whether FOXO3‐driven mitochondrial damage is associated with changes in mitophagy, we examined PINK1 and PARKIN expression in GCs by immunofluorescence. The results showed that the fluorescence intensities of both PINK1 (Figure S7A,B) and PARKIN (Figure S7C,D) were elevated in cumulus cells of the AAV‐FOXO3 group than in the AAV‐NC group. Consistently, both PINK1 (Figure S7E,F) and PARKIN (Figure S7G,H) were also elevated in the Triptolide‐treated KGN group than in the CON group. Collectively, these results indicate that FOXO3 overexpression can disrupt mitochondrial dynamic homeostasis, leading to mitochondrial fragmentation and functional decline, accompanied by enhanced PINK1/PARKIN‐mediated mitophagy signaling, suggesting that this may represent a compensatory mitochondrial quality control response to FOXO3‐driven mitochondrial damage.

### 2.8 FOXO3 Phosphorylation Inhibition and Mitochondrial Dynamic Imbalance Are Shared Features of Physiological Ovarian Aging

To determine whether the aberrant FOXO3 activation and mitochondrial dynamic imbalance identified in POI are also involved in physiological ovarian aging, we compared ovarian tissues from 2‐month‐old (2 M) and 10‐month‐old (10 M) mice.

**FIGURE 8:**
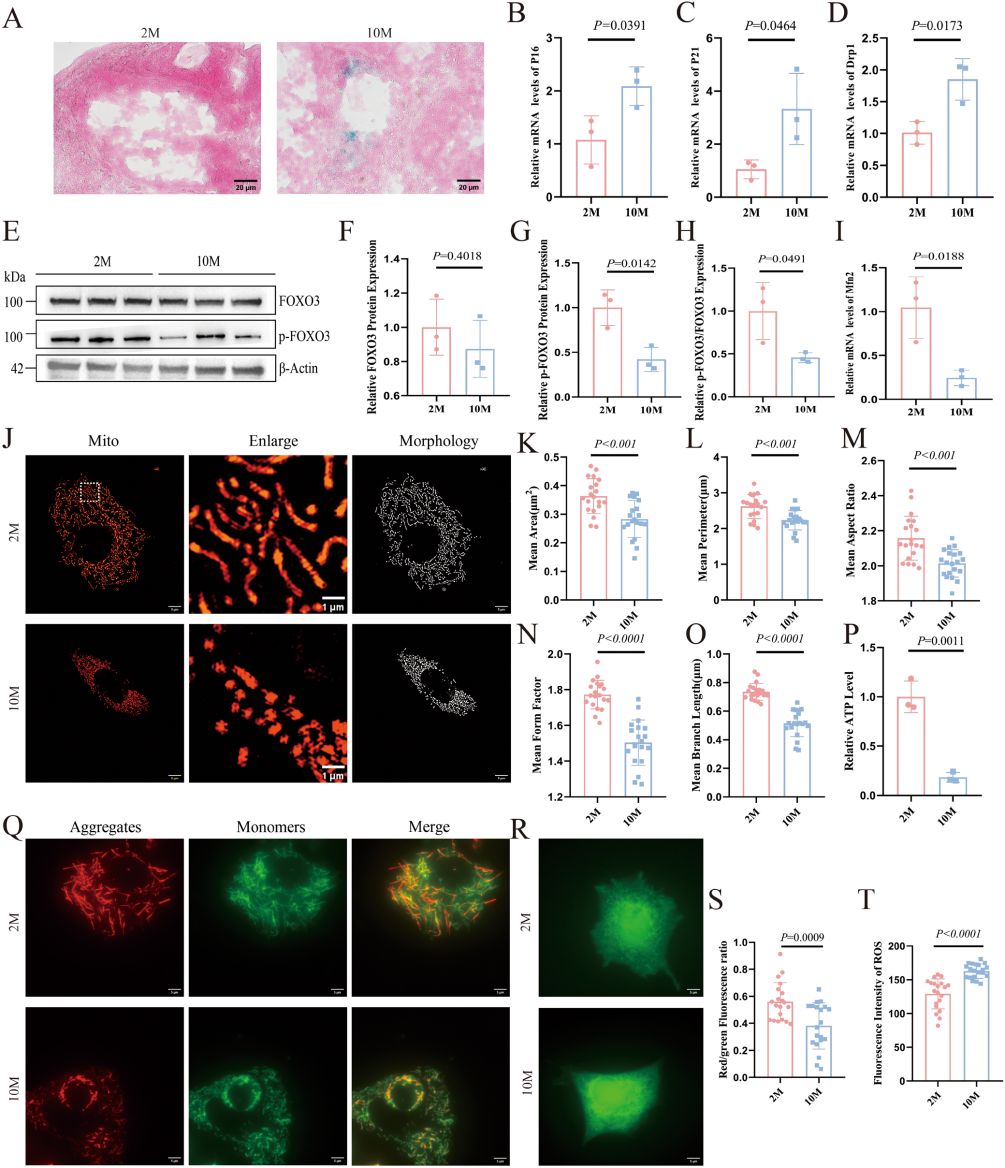
FOXO3 expression and mitochondrial dynamic imbalance during physiological ovarian aging. (A) Representative SA‐β‐gal staining images of ovarian tissues from 2‐month‐old (2 M) and 10‐month‐old (10 M) mice. Scale bar = 20 μm. (B) Relative mRNA expression level of cellular senescence marker *P16* in ovarian tissues of 2 M and 10 M mice, *n* = 3 mice per group. (C) Relative mRNA expression level of cellular senescence marker *P21* in ovarian tissues of the two groups, *n* = 3 mice per group. (D) Relative mRNA expression level of mitochondrial fission gene *Drp1* in ovarian tissues of the two groups, *n* = 3 mice per group. (E) Western blot bands of total FOXO3 and phosphorylated FOXO3 (p‐FOXO3) proteins in ovarian tissues from 2 M and 10 M mice. (F) Quantitative analysis of relative total FOXO3 protein expression, *n* = 3 mice per group. (G) Quantitative analysis of relative p‐FOXO3 protein expression, *n* = 3 mice per group. (H) Quantitative analysis of the p‐FOXO3/FOXO3 protein ratio, *n* = 3 mice per group. (I) Relative mRNA expression level of mitochondrial fusion gene *Mfn2* in ovarian tissues of the two groups, *n* = 3 mice per group. (J) Super‐resolution structured illumination microscopy images of mitochondria stained with MitoTracker in GCs from 2 M and 10 M mice, including the original field, enlarged view and morphological binarization image; Scale bar = 5 μm, inset scale bar = 1 μm. (K–O) Quantitative statistical analysis of mitochondrial mean area, mean perimeter, mean aspect ratio, mean form factor and mean branch length in granulosa cells of the two groups, *n* = 20 biological replicates. (P) Relative ATP level quantification in ovarian granulosa cells, *n* = 3 biological replicates. (Q) Representative JC‐1 staining images for mitochondrial membrane potential detection; red fluorescence indicates JC‐1 aggregates, green fluorescence indicates JC‐1 monomers; scale bar = 5 μm. (R) Representative DCFH‐DA immunofluorescence images for intracellular ROS detection (green fluorescence); scale bar = 5 μm. (S) Quantitative analysis of JC‐1 red/green fluorescence ratio, *n* = 20 biological replicates. (T) Quantitative analysis of intracellular ROS fluorescence intensity in granulosa cells, *n* = 20 biological replicates.

First, to evaluate the senescent phenotype associated with physiological ovarian aging, ovarian tissue sections from 2 M and 10 M mice were stained with SA‐β‐gal. Distinct blue‐stained SA‐β‐gal‐positive areas were observed in 10 M ovarian tissue, whereas little to no blue staining was detected in the 2 M group (Figure 8A), indicating progressive senescent cell accumulation during natural ovarian aging. Consistently, qRT‐PCR results showed that *P16* (Figure 8B) and *P21* (Figure 8C) mRNA levels were significantly elevated in 10 M ovaries, further confirming the presence of a pronounced senescent phenotype in 10 M mouse ovaries.

To further clarify changes in the FOXO3 pathway during physiological ovarian aging, western blot analysis was performed on ovarian tissues from 2 M and 10 M mice. Compared with the 2 M group, p‐FOXO3 levels were significantly reduced in 10 M ovaries (Figures 8E–H), suggesting FOXO3 pathway activation during physiological ovarian aging, consistent with the trend observed in the POI model.

In addition, *Drp1* and *Mfn2* expression was elevated and reduced in 10 M ovaries (Figure 8D,I), respectively, reflecting a pattern of excessive mitochondrial fission consistent with the mitochondrial dynamic imbalance observed in the POI model.

Super‐resolution structured illumination microscopy revealed that GCs from 2 M mice displayed elongated, interconnected mitochondrial networks, whereas those from 10 M mice exhibited markedly increased mitochondrial fragmentation (Figure 8J). Quantitative analysis confirmed that mean mitochondrial area, perimeter, form factor, aspect ratio, and branch length reduced in 10 M GCs (Figure 8K–O), along with decreased ATP levels (Figure 8P). JC‐1 staining showed a reduced red‐to‐green fluorescence ratio in 10 M GCs, indicating MMP loss (Figure 8Q,S). DCFH‐DA staining confirmed elevated ROS levels in 10 M GCs (Figure 8R,T).

Taken together, these results demonstrate that FOXO3 activation, mitochondrial dynamic homeostasis disruption, and mitochondrial functional decline are shared core features of both POI pathology and physiological ovarian aging.

## 3 Discussion

This study generated a spatial transcriptomic map of ovaries from mice with POI, revealing spatially organized ovarian abnormalities. Using this dataset, we characterized the spatial distribution and gene‐expression profiles of GCs and identified three GC subtypes with blocked differentiation trajectories, indicating early initiation of cellular aging. Mitochondrial dysfunction emerged as a central driver of this process. Consistent with previous studies, we confirmed that disruption of mitochondrial dynamic homeostasis is a major cause of mitochondrial functional impairment. We further identified FOXO3 as a key transcription regulator of mitochondrial dynamics and showed that inhibiting its overactivation can partially restore mitochondrial function in POI. These findings provide spatially resolved insight into POI pathogenesis and identify potential therapeutic targets.

We observed marked spatial disorganization in POI ovaries, characterized by reduced follicular regions and expansion of non‐follicular areas. Similar structural alterations have been reported in aging non‐human ovaries (Lu et al. 2024), suggesting that, despite differing etiologies, aging‐associated mechanisms contribute to ovarian functional decline. Spatial transcriptomic analysis indicated that this spatial disorder is associated with the developmental arrest of EGCs and depletion of CGCs, consistent with GC subtype‐specific aging observed in human ovaries (Wu et al. 2024 ).

The molecular regulation of granulosa‐cell senescence in POI remains incompletely understood. In the present study, qRT‐PCR and immunohistochemical analysis revealed significantly elevated *P16* and *P21* mRNA levels in POI GCs, with protein‐level confirmation of P21, providing direct evidence for the senescent phenotype of GCs in POI. We further identified marked mitochondrial structural and functional damage in these cells. Mitochondrial dysfunction is a key hallmark of aging (Ju et al. 2024) and leads to reduced ATP production and excessive ROS generation, thereby intensifying oxidative stress and forming a vicious cycle (Acin‐Perez et al. 2023; Chen et al. 2024). This dysfunction is closely linked to impaired mitochondrial dynamics, a critical process for maintaining mitochondrial quality control and network integrity (Shang et al. 2025; Amartuvshin et al. 2020). Dysregulated mitochondrial dynamics have been implicated in multiple age‐related disorders and reproductive aging. Here, both in vitro and in vivo data showed that GCs in POI exhibit excessive mitochondrial fission and reduced fusion, indicating a collapse of mitochondrial dynamic balance that promotes cellular senescence.

We further demonstrated that FOXO3 is a central transcriptional regulator of mitochondrial dynamics in GCs. During POI progression, FOXO3 was aberrantly activated, and inhibiting this aberrant activation alleviated mitochondrial dynamic imbalance and partially restored mitochondrial function and structure (Yao et al. 2022). FOXO3 regulates multiple genes involved in mitochondrial autophagy, biosynthesis, and fission–fusion balance and has been widely implicated in cellular senescence (Cao et al. 2023). These pharmacological intervention experiments were performed in KGN cells, a transformed human granulosa‐like tumor cell line that may differ from primary murine GCs in metabolic state and stress responsiveness; therefore, the effects of JY‐2 and ipatasertib in primary GC systems warrant further investigation. To further validate the relationship between FOXO3 and mitochondrial dynamics, we delivered AAV‐FOXO3 via intraovarian injection to overexpress FOXO3 in primary mouse granulosa cells in vivo. Critically, FOXO3 overexpression was sufficient to induce a senescent phenotype in granulosa cells, as evidenced by marked upregulation of *P16* and *P21* mRNA levels and increased SA‐β‐gal positivity in ovarian tissue sections. Moreover, FOXO3 overexpression recapitulated excessive mitochondrial fission and functional decline, consistent with observations in the cell model and providing in vivo support for the above conclusions. In addition, PINK1 and PARKIN expression was elevated in both the in vivo FOXO3 overexpression and triptolide‐induced KGN cell models, suggesting that FOXO3‐driven mitochondrial damage may be accompanied by enhanced mitophagy through the PINK1/PARKIN pathway, consistent with previous reports on FOXO3‐mediated regulation of mitophagy (Yao et al. 2022). Our findings identify FOXO3 as a promising target for improving GC mitochondrial function and delaying ovarian aging, consistent with previous regulatory models (Li et al. 2022).

To determine whether the FOXO3–mitochondrial axis is also involved in natural reproductive senescence, we compared ovaries from 2 M and 10 M mice. We found that physiologically aged ovaries (10 M) closely recapitulated the pathological features observed in the triptolide‐induced POI model. Specifically, 10 M ovaries exhibited robust FOXO3 activation—evidenced by diminished p‐FOXO3 levels—alongside the upregulation of senescence markers p16 and p21. These alterations were coupled with a profound disruption of mitochondrial dynamic homeostasis, characterized by DRP1 upregulation and MFN2 downregulation, leading to extensive mitochondrial fragmentation and functional failure (manifested by reduced ATP production, loss of mitochondrial membrane potential, and elevated ROS levels). These findings suggest that FOXO3‐mediated mitochondrial dynamic imbalance is potentially a conserved feature of ovarian functional decline, rather than a specific byproduct of exogenous drug‐induced injury.

In summary, this study constructed a spatial transcriptomic atlas of POI and found that FOXO3 activation may contribute to GC senescence by disrupting mitochondrial dynamic homeostasis, thereby promoting ovarian functional decline. Similar findings in physiologically aging ovaries suggest that targeting FOXO3‐related pathways may be a viable intervention for ovarian aging. Since in vivo rescue experiments have not yet been performed, the therapeutic implications remain preliminary and await further validation. These findings provide a new perspective for understanding POI pathogenesis and offer a reference for future studies.

## 4 Materials and Methods

### 4.1 Animal Model and Ethical Approval

Eight‐week‐old female C57BL/6 mice were purchased from Jiangsu Qinglongshan Biotechnology Co. Ltd. After a 3‐day acclimatization period, mice were randomly assigned to the CON or POI group. POI was induced by intragastric administration of tripterygium glycosides (75 mg/kg/day, dissolved in 0.9% saline) for 14 consecutive days following a previously established protocol (Luo et al. 2026), whereas CON mice received an equal volume of saline.

For the AAV‐FOXO3 overexpression assay and physiological ovarian aging comparison between 2‐month‐old and 10‐month‐old mice, ovarian tissues and cumulus‐oocyte complexes (COCs) from separate batches of animals within each group were harvested for distinct downstream examinations, including western blot, ATP quantification, qRT‐PCR, super‐resolution microscopic assessment of mitochondrial morphology, ROS and JC‐1 mitochondrial membrane potential detection, as well as immunofluorescence quantification targeting DRP1, MFN2, FOXO3, and phosphorylated FOXO3.

The mice were randomly allocated to each experimental group via a random number table by a researcher who did not take part in subsequent experiments. All image acquisition and quantitative analyzes were completed by investigators blinded to group assignments. Microscope parameters were standardized across all groups, and images were captured from randomly selected fields of view, with group labels hidden. Fluorescence intensity measurement and morphological analysis were carried out on images marked with random anonymous identifiers; group information was only unblinded after all quantitative data analysis.

All procedures were approved by the Animal Ethics Committee of Nanjing University of Chinese Medicine (approval no. 202404A031).

### 4.2 Spatial Transcriptomic Analysis Using 10× Genomics Xenium

Ovarian tissues were harvested from control (CON) and POI model mice (*n* = 3 per group). All sample testing was conducted by the authorized service provider, LC‐Bio Technology Co. Ltd. (Hangzhou, China), on the 10× Genomics in situ gene expression platform. Targeted transcriptional profiling was performed with the Xenium Mouse 5 K Panel, and all experimental operations strictly followed the manufacturer's standard protocols. FFPE sections underwent deparaffinization and decrosslinking, followed by overnight hybridization with gene‐specific probes. After ligation and rolling‐circle amplification (RCA), multi‐cycle fluorescence imaging was carried out on the Xenium Analyzer. Cell segmentation was implemented via the instrument's built‐in workflow combining three strategies: boundary marker staining (ATP1A1/CD45/E‐cadherin), intracellular RNA labelling (18S), and 5.0 μm outward expansion of nuclear regions.

Raw data processing, including transcript decoding, deduplication, and single‐cell segmentation, was completed by the native analytical pipeline of the Xenium instrument. This platform enables parallel detection of all tissue sections on a single chip; however, independent quality control metrics for individual samples cannot be exported from the equipment. Accordingly, this study reports the global QC parameters of the entire sequencing run. A total of 544,026 cells were identified across all sections, with a median of 423 detected transcripts and 331 unique genes per cell. Transcripts localized within cell contours accounted for 96.5% detected signals, while blank signal‐free spots only made up 0.1%, verifying the high reliability of single‐cell segmentation.

Secondary quality filtering was performed in Python using the Scanpy and Seurat toolkits, with all unspecified parameters set to their default values. Cells with fewer than 50 total detected transcripts (sc.pp.filter_cells (adata, min_counts = 50)) and genes detected in fewer than 50 cells (sc.pp.filter_genes (adata, min_cells = 50)) were discarded. High‐quality cells retained after filtering were subjected to subsequent bioinformatic analyzes.

Datasets from six samples (3 CON, 3 POI) were integrated, and raw count matrices were preserved prior to normalization (adata.layers[“counts”] = adata.X.copy()). Gene expression values were normalized via Scanpy's standard total‐count normalization followed by log1p transformation (sc.pp.normalize_total, sc.pp.log1p). Principal component analysis (sc.pp.pca), nearest‐neighbor graph construction (sc.pp. neighbors), and UMAP dimensionality reduction embedding (sc.tl.umap) were performed with default settings. Unsupervized clustering was achieved using the Leiden algorithm (sc.tl.leiden, default resolution = 1.0), yielding 38 transcriptionally distinct subclusters, which were annotated into nine major cell types based on canonical marker genes listed in Table S1 Pseudotime trajectory analysis was performed using Monocle3, and in situ spatial gene expression maps were visualized with Xenium Explorer software.

Differentially expressed genes (DEGs) between granulosa cells of the control (CON) and premature ovarian insufficiency (POI) groups were identified by LC‐Bio Technology Co. Ltd. using the Wilcoxon rank‐sum test in Scanpy (function: sc.tl.rank_genes_groups, method = “wilcoxon”), based on the full Xenium Mouse 5 K probe library containing 5002 genes (Table S2). The Benjamini–Hochberg method was used for multiple testing correction to control the false discovery rate (FDR). Genes with *p* < 0.05 were defined as DEGs. The CytoTrace algorithm was applied to assess cell differentiation status, and differentiation scores derived from spatial transcriptomic data were projected onto UMAP plots for visualization.

The above DEGs were intersected with the mouse cellular senescence gene set retrieved from the Aging Atlas database, generating 339 overlapping genes. Among them, 221 genes exhibited statistically significant expression changes (*p* < 0.05), comprising 88 upregulated genes and 133 downregulated genes, which were selected as senescence‐related candidate genes for downstream analyzes. Gene Ontology (GO) and KEGG pathway enrichment analyzes of the intersecting gene set were conducted using the DAVID database.

A protein–protein interaction (PPI) network of overlapping genes was constructed using the STRING database, with a minimum interaction confidence threshold of 0.7. The PPI network was visualized using Cytoscape 3.10, and hub genes were identified based on node degree values.

### 4.3 Transmission Electron Microscopy

Left ovaries from three randomly selected mice per group were fixed in 2.5% glutaraldehyde, followed by post‐fixation with osmium tetroxide, dehydration, epoxy‐resin infiltration, and embedding. Semi‐thin sections were stained with toluidine blue for orientation, and ultra‐thin sections were prepared and stained with lead citrate. Mitochondrial ultrastructure in ovarian GCs was examined using a transmission electron microscope.

### 4.4 Histological Analysis and Follicle Counting

Ovarian tissues were fixed in 4% PFA, embedded in paraffin, and serially sectioned at a thickness of 4 μm. Every sixth section was stained with HE, yielding six representative planes per ovary. Ovarian morphology and follicles at various developmental stages were counted under a light microscope.

### 4.5 Senescence‐Associated β‐Galactosidase (SA‐β‐Gal) Staining

The senescence‐associated β‐galactosidase (SA‐β‐gal) staining kit (Beyotime, C0602) was used for detection. After washing 10 μm frozen sections three times with PBS, the sections were fixed in SA‐β‐gal fixative solution at room temperature for 15 min. Following thorough PBS rinsing, an appropriate volume of staining working solution was added, and incubation was performed overnight at 37°C. Nuclei were counterstained with Nuclear Fast Red staining solution (Beyotime, C0151), and the sections were mounted with neutral balsam. Bright‐field light microscopy at 400× magnification was used to observe and image blue‐stained SA‐β‐gal‐positive regions.

### 4.6 Immunohistochemistry

Paraffin sections were deparaffinized, rehydrated, and incubated with 3% H_2_O_2_ for 10 min to block endogenous peroxidase activity. After washing with phosphate‐buffered saline (PBS), antigen retrieval was performed in sodium citrate buffer (pH 6.0) under pressure. Sections were blocked with 5% bovine serum albumin (BSA) for 30 min and incubated overnight at 4°C with the following primary antibodies: anti‐FOXO3 (Huabio, ET1604‐11, 1:100), anti‐p‐FOXO3 (Huabio, ET1609‐49, 1:100), anti‐DRP1 (Proteintech, 12957‐1‐AP, 1:200), anti‐MFN2 (Proteintech, 67487‐1‐IG, 1:2000), anti‐MFN1 (Proteintech, 13798‐1‐AP, 1:200), anti‐OPA1 (Proteintech, 66583‐1‐Ig, 1:600), anti‐MFF (Proteintech, 17090‐1‐AP, 1:1000), anti‐P21 (Abmart, T55543S, 1:400). Detection was performed using an SABC Kit (BOSTER, SA1020). Sections were incubated with biotinylated goat anti‐mouse/rabbit IgG secondary antibody for 1 h at room temperature, washed with PBS, incubated with SABC reagent for 30 min, developed with DAB, and counterstained with hematoxylin. Six random fields of view per section were imaged at 400× magnification, and the MOD of target proteins was calculated using ImageJ FIJI.

### 4.7 Isolation of Ovarian GCs and Cumulus‐Oocyte Complexes (COCs)

#### 4.7.1 GCs

Bilateral ovaries were placed in ice‐cold PBS. Follicles were punctured under a stereomicroscope using syringe needles to release GCs. The suspension was passed through a 200‐mesh cell strainer, centrifuged at 1000 rpm for 5 min, and the pellet was resuspended in PBS and adjusted to a density of 1 × 10^6^ cells/mL for subsequent use.

#### 4.7.2 COCs

Mice were superovulated by intraperitoneal injection of 10 IU pregnant mare serum gonadotropin (PMSG), followed 48 h later by 10 IU human chorionic gonadotropin (hCG). After 14 h, ovaries and oviducts were collected in PBS. COCs were released by tearing the oviductal ampulla under a stereomicroscope, collected by centrifugation at 1000 rpm for 5 min, and resuspended in DMEM/F12 medium.

To compare the CON and POI groups, COCs from each mouse (*n =* 6 per group) were processed separately, with each data point representing one animal. For AAV‐FOXO3 overexpression and physiological aging (2 M vs. 10 M) comparisons, COCs from 6 mice per group were pooled and divided into 3 or 4 aliquots for different downstream assays. All GC isolations across experimental groups were performed in parallel on the same day to minimize inter‐batch variability. Cell viability was assessed by trypan blue exclusion prior to all downstream assays; representative viability data are provided in Figure S3F,G.

### 4.8 Mitochondrial Mass Detection (MitoTracker Red Staining)

COCs isolated from CON and POI mice were incubated with 200 nM MitoTracker Red CMXRos working solution for 20 min, centrifuged at 1000 × g for 5 min, and the supernatant was discarded. After washing with cell culture medium, nuclei were counterstained with Hoechst 33342 (Biosharp, BL803A) for 5 min. Following another wash, samples were mounted and examined under a fluorescence microscope. Each data point represents the mean fluorescence intensity from one mouse.

### 4.9 ATP Content Measurement

ATP levels were measured using an ATP Assay Kit (Beyotime, S0026). Cells or tissues were lysed in ATP lysis buffer, and the supernatant was mixed with ATP detection reagent. Fluorescence was measured using a multifunctional microplate reader (PerkinElmer, EnVision), and ATP concentrations were calculated based on a standard curve.

For comparing CON and POI groups, the GCs were collected from individual mice (*n =* 6 per group); each data point represents one mouse. For AAV‐FOXO3 overexpression and physiological aging (2 M vs. 10 M) comparisons, bilateral ovaries (after COC retrieval) from individual mice were used (*n =* 3 per group); each data point represents one mouse. For KGN cell experiments, ATP quantification was performed across *n =* 3 independent experimental replicates; each data point represents one independent experiment.

### 4.10 Mitochondrial Membrane Potential Assay (JC‐1 Staining)

MMP was assessed using a JC‐1 Assay Kit (Beyotime, C2003s).

For comparing CON and POI mice, GCs isolated from individual mice were incubated with JC‐1 working solution at 37°C for 20 min. The red‐to‐green fluorescence ratio (590/530 nm) was measured with a flow cytometer (Beckman Gallios), and data were analyzed with FlowJo v10.9. Each data point represents one mouse (*n* = 6 per group).

For COCs harvested from AAV‐FOXO3, AAV‐NC, 2‐month‐old, and 10‐month‐old mice, cumulus cells were dissociated from pooled COC preparations (derived from 4 mice per group) by repeated gentle pipetting, washed with PBS, seeded onto glass‐bottom dishes, and cultured for 24 h. The cells were incubated with JC‐1 working solution at 37°C for 20 min, and after washing, the images were captured under a super‐resolution structured illumination microscope. The red‐to‐green fluorescence ratio was quantified using ImageJ FIJI software at the single‐cell level; *n =* 20 cells per group were analyzed, and each data point represents one individual cell.

For KGN cells, JC‐1 staining was performed identically; fluorescence was quantified per microscopic field of view (*n =* 8 fields per group), and each data point represents one field of view.

For all JC‐1 experiments, the working solution was prepared at a consistent concentration across experimental sessions. A carbonyl cyanide m‐chlorophenyl hydrazone (CCCP)‐treated positive control group was included to confirm probe functionality and validate the expected shift from red to green fluorescence upon MMP dissipation. Image acquisition parameters, including laser power and exposure time, were constant across all groups within each experiment, and unnecessary light exposure was minimized throughout the staining and imaging procedure to reduce photobleaching. The red‐to‐green fluorescence ratio was used as the quantitative readout, which inherently accounts for cell‐to‐cell variation in dye uptake. For flow cytometric analysis, gating strategies and the number of valid cell events recorded per sample are illustrated in Figure 3M.

### 4.11 Reactive Oxygen Species (ROS) Detection

ROS levels were determined using a ROS assay kit (Nanjing Jiancheng, E004‐1‐1).

The COCs isolated from individual CON and POI mice were incubated with 10 μM DCFH‐DA working solution at 37°C for 20 min in the dark. After washing, the COCs were stained with Hoechst 33342 and observed under a fluorescence microscope. Each data point represents the mean fluorescence intensity from one mouse (*n =* 6 per group).

For COCs from AAV‐FOXO3, AAV‐NC, 2‐month‐old, and 10‐month‐old mice, cumulus cells were dissociated from pooled COC preparations (derived from 4 mice per group) by pipetting, washed with PBS, seeded onto glass‐bottom dishes, and cultured for 24 h. Cells were incubated with 10 μM DCFH‐DA (37°C, 20 min, dark), counterstained with Hoechst 33342, and imaged by super‐resolution SIM. Fluorescence intensity was quantified at the single‐cell level using ImageJ FIJI; *n* = 20 cells per group were analyzed, and each data point represents one individual cell.

For KGN cells, ROS detection was performed under identical conditions and quantified per microscopic field of view (*n* = 10 fields per group); each data point represents one field of view.

### 4.12 Quantitative Reverse Transcription‐Polymerase Chain Reaction (qRT‐PCR)

Total RNA was isolated from GCs with the Hifair III 1st Strand cDNA Synthesis Kit (Yeasen, Cat. No.: 11141ES60). Quantitative real‐time polymerase chain reaction (qRT‐PCR) assays were conducted on a quantitative PCR detection system (Molarray, Model: MA‐6000), employing the Hieff qPCR SYBR Green Master Mix (Low Rox Plus) (Yeasen, Cat. No.: 11202ES08). The *Gapdh* gene served as the internal reference, and the relative mRNA expression levels of target genes were quantified via the 2^−ΔΔCT^ method.

### 4.13 Cell Culture and In Vitro Modeling

The human granulosa‐like tumor cell line KGN was cultured in DMEM/F12 medium (Zhongqiao Xinzhou, ZQ‐600) supplemented with 10% FBS and 1% penicillin–streptomycin at 37°C in a humidified incubator with 5% CO_2_.

#### 4.13.1 POI Model

KGN cells were treated with 0.05 μM triptolide (MCE, HY‐32735) for 24 h.

#### 4.13.2 FOXO3 Overexpression Model

KGN cells were treated with 30 μM ipatasertib (MCE, HY‐15186) for 24 h.

#### 4.13.3 FOXO3 Inhibition Model

KGN cells were pretreated with 0.01 μM JY‐2 (MCE, HY‐153347) for 12 h, washed, and then treated with 0.05 μM triptolide for an additional 24 h.

### 4.14 Mitochondrial Membrane Potential Detection (PK Mito Orange Staining)

MMP was measured using the PK Mito Orange fluorescent probe (GenVivo, PKMO‐2). KGN cells were incubated with the working solution at 37°C for 15 min, washed with culture medium, and observed under a super‐resolution microscope. Fluorescence intensity was quantified using ImageJ FIJI.

For COCs from AAV‐FOXO3, AAV‐NC, 2‐month‐old, and 10‐month‐old mice, cumulus cells were dissociated, washed with PBS, seeded onto glass‐bottom dishes, and cultured for 24 h. After PK Mito Orange staining (37°C, 15 min), super‐resolution images were acquired, and mitochondrial morphology was quantified as described above.

### 4.15 Cell Immunofluorescence

KGN cells were fixed with 4% PFA for 15 min, permeabilized with 0.1% Triton X‐100 for 15 min, and blocked with 5% BSA for 30 min. Cells were incubated overnight at 4°C with the following primary antibodies: anti‐FOXO3 (1:100), anti‐p‐FOXO3 (1:100), anti‐DRP1 (1:100), anti‐MFN2 (1:100), anti‐PINK1 (Proteintech, 23274‐1‐ap, 1:100), and anti‐PARKIN (Wanleibio, 1:100). After washing with PBST, cells were incubated with corresponding fluorescent secondary antibodies (Beyotime, A0423/A0428) for 1 h at room temperature. Nuclei were counterstained with DAPI (Biosharp, BL105A) for 5 min, and samples were observed under a super‐resolution microscope. Fluorescence intensity was quantified at the single‐cell level; *n =* 20 cells per group were analyzed, and each data point represents one individual cell.

### 4.16 Super‐Resolution Microscopy and Image Analysis

Super‐resolution microscopy was performed with the assistance of NanoInsights‐Tech Co. Ltd. (Beijing, China).

#### 4.16.1 Multi‐SIM Super‐Resolution Microscopy

Imaging was performed using a multi‐SIM system equipped with a 100X/1.49 NA oil immersion objective, semiconductor/solid‐state lasers (405, 488, 561, and 640 nm), and an sCMOS camera. Immersion oil with a refractive index of 1.518 was used. The system was routinely calibrated with 100 nm fluorescent beads. SIM image stacks were reconstructed using SI‐Recon software (v2.23.3) with the following parameters: pixel size 30.6 nm; channel‐specific optical transfer functions; Wiener filter constant (0.01 for 2D mode, 0.005 for 3D mode); and negative intensity background. Total variation (TV) denoising was applied after reconstruction.

#### 4.16.2 Deconvolution Microscopy

Wide‐field image stacks were processed in SI‐Recon using Richardson‐Lucy or HVL1 deconvolution. Channel registration was corrected to within 1 pixel using 100 nm fluorescent beads.

### 4.17 Western Blotting

Total cellular proteins were extracted using RIPA lysis buffer (NCM Biotech, WB3100) supplemented with protease and phosphatase inhibitors (Biosharp, BL1439A). Protein samples were separated by SDS‐PAGE electrophoresis, transferred onto PVDF membranes, and blocked with QuickBlock Protein‐free Western Blocking Buffer (Beyotime, P0240) for 30 min. The membranes were individually incubated with the following primary antibodies overnight at 4°C on a shaker: anti‐FOXO3 (Huabio, ET1604‐11, 1:2000), anti‐FOXO3 (Wanleibio,WL02891,1:1000), anti‐p‐FOXO3 (Huabio, ET1609‐49, 1:1000), and anti‐β‐actin (Proteintech, 66009‐1‐Ig, 1:1000) as a loading control. After secondary antibody incubation the next day, protein bands were visualized using a chemiluminescence imaging system. In addition, some membranes were stripped no more than once (NCM Biotech, WB6200) to detect more proteins of interest.

### 4.18 AAV Vector Construction and Intraovarian Injection

Recombinant adeno‐associated virus vectors overexpressing murine Foxo3 (AAV‐FOXO3) and empty vector negative control (AAV‐NC) were constructed and packaged by GenePharma Co. Ltd. The viral serotype was AAV9 with a titer of 1.99 × 10^13^ vg/mL (vector genomes per milliliter).

Eight‐week‐old female C57BL/6 mice were anesthetized via isoflurane inhalation. A dorsal flank incision was made to surgically expose bilateral ovaries. Using a microsyringe, 2 μL AAV‐FOXO3 or AAV‐NC viral solution was injected into the ovarian bursa of each ovary. The peritoneum and skin were sutured in layers with absorbable sutures. Mice were maintained under standard housing conditions for a 2‐week recovery period before tissue collection. Western blotting and tissue immunofluorescence staining were performed to confirm successful in vivo FOXO3 overexpression.

### 4.19 Statistical Analysis

Statistical analyzes were performed using SPSS version 26.0. Data are presented as mean ± standard deviation. Normality of data distribution was assessed using the Shapiro–Wilk test. For normally distributed data, two‐group comparisons were performed using unpaired two‐tailed Student's *t*‐test, and multiple‐group comparisons using one‐way analysis of variance (ANOVA). Non‐normally distributed data were analyzed using the Kruskal‐Wallis test. A *p*‐value < 0.05 was considered statistically significant unless otherwise specified.

## Author Contributions

Meihong Shen, Hongxiao Li, and Yaoli Yin conceived the project and designed the study. Ziwei Song, Meilin Chen, Xiaolu Jin, and Zemin Li performed the experiments. Ziwei Song and Xiaolu Jin analyzed the data. Ziwei Song drafted the manuscript. Meihong Shen, Hongxiao Li, and Yaoli Yin revised the manuscript. Meihong Shen and Hongxiao Li supervised the project. All authors read and approved the final manuscript.

## Funding

This work was supported by National Natural Science Foundation of China (Grant 82374599,82505778) and Jiangsu Province Leading Talents Cultivation Project for Traditional Chinese Medicine (Grant SLJ0316).

## Ethics Statement

All animal experiments were approved by the Animal Ethics Committee of Nanjing University of Chinese Medicine (Approval No. 202404A031) and were conducted in accordance with institutional and national guidelines and the ARRIVE guidelines.

## Conflicts of Interest

The authors declare no conflicts of interest.

## Supporting information


**Figure S1:** (A) Spatial localization maps of cellular structure and components in the CON and POI groups. The DAPI channel (blue) shows nuclear distribution; the Boundary channel (yellow) labels signal in cell boundary regions; the Interior‐RNA channel (pink) labels intracellular RNA localization; the Interior‐Protein channel (cyan) labels intracellular protein localization. (B) GO enrichment analysis of the top 50 marker genes in Endothelial cells, Epithelial cells, Luteal cells, Macrophages, Stromal cells, T cells, and Theca cells. (C) Follicle counts in the CON and POI groups, *n* = 6 mice per group.
**Figure S2:** (A) Monocle3 pseudotime trajectory analysis of GCs, where nodes and connecting lines represent the trajectory branches of cell populations. (B) Bar chart of CytoTrace scores for the three GC subtypes. (C) Spatial expression mapping of pro‐senescence factors *Trp53*, *Rb1* and anti‐senescence factors *Tert*, *Sod2* in ovarian tissues of the CON and POI groups. White indicates cell nuclei, purple indicates *Trp53*, red indicates *Rb1*, blue indicates *Tert*, and green indicates *Sod2*. Scale bar = 200 μm.
**Figure S3:** (A) Schematic diagram of POI mouse model establishment. C57BL/6J mice were acclimatized for 3 days, followed by vaginal exfoliative cell smearing for 10 consecutive days. Female mice with two complete estrous cycles were enrolled in the experiment. Mice in the POI group were intragastrically administered with tripterygium glycosides (TGs), while those in the CON group were given an equal volume of normal saline. Samples were collected after 14 days of intervention, and GCs and cumulus‐oocyte complexes (COCs) were isolated. (B) Representative line chart of estrous cycles. (C) Rate of estrous cycle disorders, *n* = 6 mice per group. (D) Bar charts of ovarian weight comparison (left panel), *n* = 6 mice per group; and ovarian index comparison (right panel), *n* = 6 mice per group. Ovarian index (‰) = ovarian weight (mg)/body weight of mice (g) × 1000‰. (E) Comparison of serum sex hormones (AMH, E_2_, FSH, LH, T), *n* = 6 mice per group.(F) Representative trypan blue staining image of isolated mouse ovarian granulosa cells. (G)Statistical bar chart of granulosa cell viability from three batches of COCs(COC1,COC2,COC3) via trypan blue staining.White bars stand for live cells,while black bars represent dead cells.
**Figure S4:** (A) Representative immunohistochemical images of mitochondrial fission‐related proteins (DRP1, MFF) and fusion‐related proteins (MFN2, MFN1, OPA1) in ovarian sections of the control (CON) and POI groups, with a blank control section shown on the right; scale bar =200 μm. (B) Immunohistochemical staining for total FOXO3 and phosphorylated p‐FOXO3 in ovarian tissues of the CON and POI groups; scale bar =200 μm. (C) Representative immunohistochemical images of the cellular senescence marker P21 in ovarian sections from the CON and POI groups; scale bar =200 μm.
**Figure S5:** (A) Cell viability of Triptolide at different concentrations in KGN cells. (B) Representative bands of p‐FOXO3 and FOXO3. (C) Quantitative statistical analysis of ralative FOXO3 phosphorylation levels measured as p‐FOXO3 normalized to total FOXO3 in CON group and triptolide group.
**Figure S6:** (A) Cell viability of Ipatasertib at different concentrations in KGN cells. (B) Cell viability of combined treatment with JY‐2 and Triptolide at different concentrations in KGN cells. (C) Representative bands of p‐FOXO3 and FOXO3. The lanes are arranged from left to right as follows: CON group, Triptolide group, Ipatasertib group and JY‐2 group. (D) Statistical analysis of WB gray values.
**Figure S7:** (A) Representative immunofluorescence images of PINK1 in ovarian cumulus cells from the AAV‐NC group and AAV‐FOXO3 group. Nuclei were stained with Hoechst. Scale bar = 5 μm.(B) Quantitative analysis of PINK1 fluorescence intensity in the AAV‐NC group and AAV‐FOXO3 group. (C) Representative immunofluorescence images of PARKIN in ovarian cumulus cells from the AAV‐NC group and AAV‐FOXO3 group. Nuclei were stained with Hoechst.Scale bar = 5 μm. (D) Quantitative analysis of PARKIN fluorescence intensity in the AAV‐NC group and AAV‐FOXO3 group. (E) Representative immunofluorescence images of PINK1 in KGN cells of the CON group and triptolide‐treated group. Nuclei were counterstained with Hoechst.Scale bar = 5 μm. (F) Quantitative analysis of PINK1 fluorescence intensity in the CON group and triptolide‐treated group. (G) Representative immunofluorescence images of PARKIN in KGN cells of the CON group and triptolide‐treated group. Nuclei were counterstained with Hoechst.Scale bar = 5 μm. (H) Quantitative analysis of PARKIN fluorescence intensity in the CON group and triptolide‐treated group.

**Table S1:** Canonical ovarian cell marker genes.

**Table S2:** Xenium Mouse 5 K probe library.

## Data Availability

The raw Xenium spatial transcriptomics data and processed gene expression matrices generated in this study have been deposited in the NCBI Gene Expression Omnibus (GEO) under accession number [GSE334364] and will be publicly accessible upon manuscript acceptance. All other data generated or analyzed during this study are included in this published article and its Supporting Information files. The analytical workflow parameters are fully documented in Section 4.2 of the Materials and Methods.
