# Supplementary material for: Spatial Transcriptomic Atlas Reveals That Forkhead Box O3‐Mediated Mitochondrial Dynamics Imbalance Drives Premature Ovarian Insufficiency in Mice
Source: Aging Cell. 2026 Jul 11;25(7):e70623. doi: 10.1111/acel.70623 (PMC13355553; doi:10.1111/acel.70623)
Supplement: Supplementary file 1 — Figure S1: (A) Spatial localization maps of cellular structure and components in the CON and POI groups. The DAPI channel (blue) shows nuclear distribution; the Boundary channel (yellow) labels signal in cell boundary regions; the Interior‐RNA channel (pink) labels intracellular RNA localization; the Interior‐Protein channel (cyan) labels intracellular protein localization. (B) GO enrichment analysis of the top 50 marker genes in Endothelial cells, Epithelial cells, Luteal cells, Macrophages, Stromal cells, T cells, and Theca cells. (C) Follicle counts in the CON and POI groups, n = 6 mice per group. Figure S2: (A) Monocle3 pseudotime trajectory analysis of GCs, where nodes and connecting lines represent the trajectory branches of cell populations. (B) Bar chart of CytoTrace scores for the three GC subtypes. (C) Spatial expression mapping of pro‐senescence factors Trp53, Rb1 and anti‐senescence factors Tert, Sod2 in ovarian tissues of the CON and POI groups. White indicates cell nuclei, purple indicates Trp53, red indicates Rb1, blue indicates Tert, and green indicates Sod2. Scale bar = 200 μm. Figure S3: (A) Schematic diagram of POI mouse model establishment. C57BL/6J mice were acclimatized for 3 days, followed by vaginal exfoliative cell smearing for 10 consecutive days. Female mice with two complete estrous cycles were enrolled in the experiment. Mice in the POI group were intragastrically administered with tripterygium glycosides (TGs), while those in the CON group were given an equal volume of normal saline. Samples were collected after 14 days of intervention, and GCs and cumulus‐oocyte complexes (COCs) were isolated. (B) Representative line chart of estrous cycles. (C) Rate of estrous cycle disorders, n = 6 mice per group. (D) Bar charts of ovarian weight comparison (left panel), n = 6 mice per group; and ovarian index comparison (right panel), n = 6 mice per group. Ovarian index (‰) = ovarian weight (mg)/body weight of mice (g) × 1000‰. (E) Comparison of ser [file ACEL-25-e70623-s002.docx]

**Supporting information**

**Figure S1**


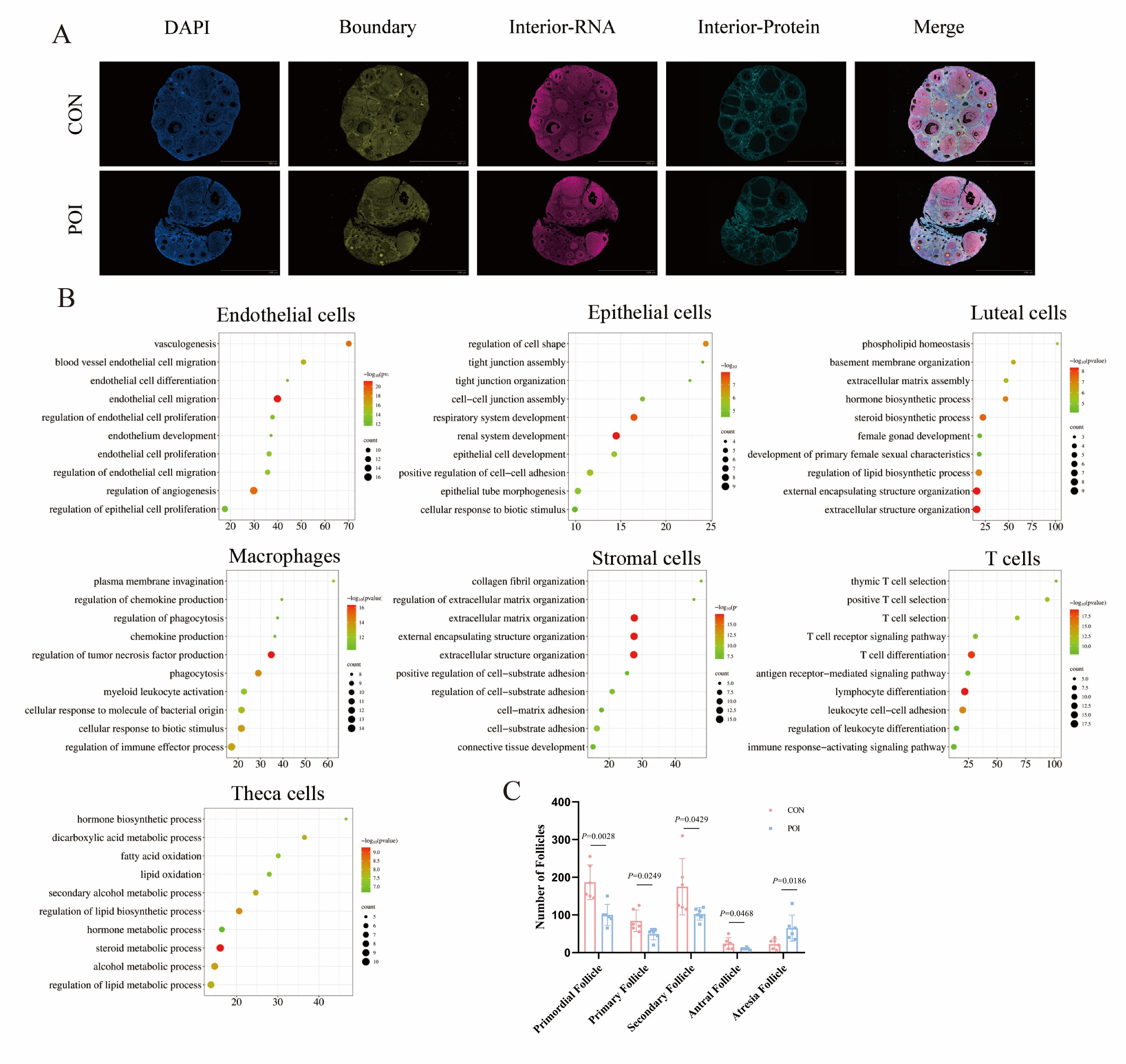


**Figure S1:** (A) Spatial localization maps of cellular structure and components in the CON and POI groups. The DAPI channel (blue) shows nuclear distribution; the Boundary channel (yellow) labels signal in cell boundary regions; the Interior‐RNA channel (pink) labels intracellular RNA localization; the Interior‐Protein channel (cyan) labels intracellular protein localization. (B) GO enrichment analysis of the top 50 marker genes in Endothelial cells, Epithelial cells, Luteal cells, Macrophages, Stromal cells, T cells, and Theca cells. (C) Follicle counts in the CON and POI groups, *n* = 6 mice per group.

**Figure S2**


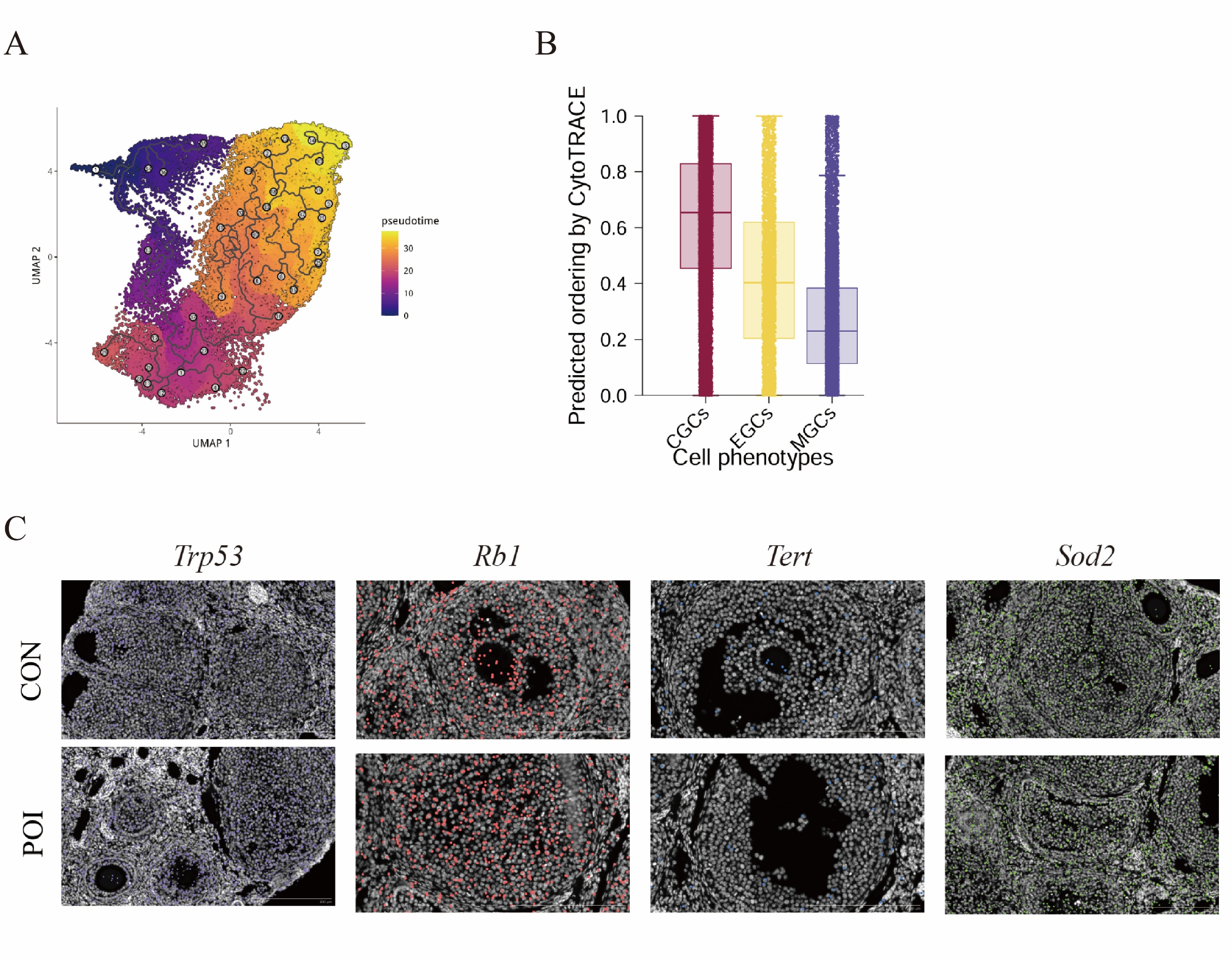


**Figure S2:**(A) Monocle3 pseudotime trajectory analysis of GCs, where nodes and connecting lines represent the trajectory branches of cell populations. (B) Bar chart of CytoTrace scores for the three GC subtypes. (C) Spatial expression mapping of pro‐senescence factors *Trp53*, *Rb1* and anti‐senescence factors *Tert*, *Sod2* in ovarian tissues of the CON and POI groups. White indicates cell nuclei, purple indicates *Trp53*, red indicates *Rb1*, blue indicates *Tert*, and green indicates *Sod2*. Scale bar = 200 μm.

**Figure S3**


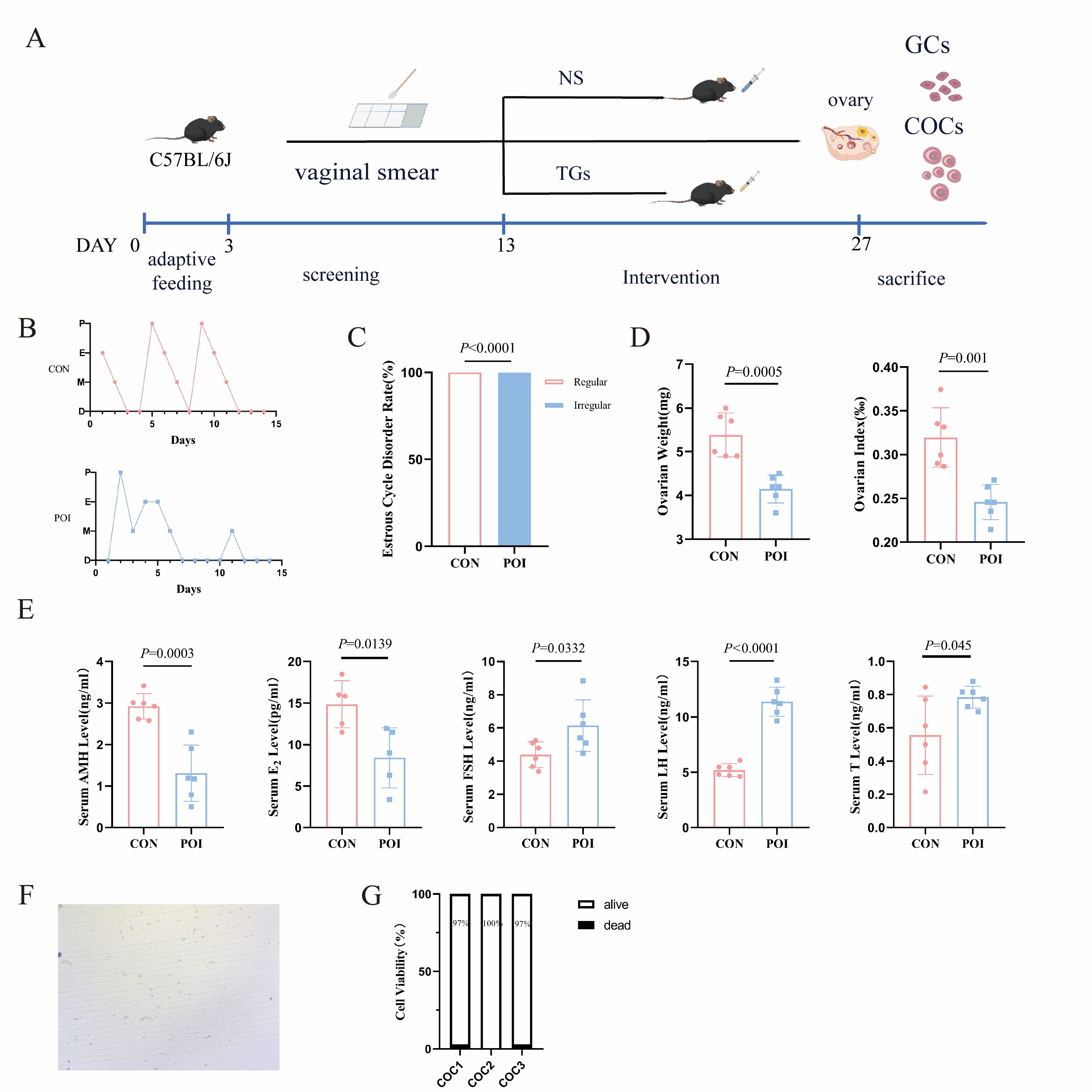


**Figure S3:** (A) Schematic diagram of POI mouse model establishment. C57BL/6J mice were acclimatized for 3 days, followed by vaginal exfoliative cell smearing for 10 consecutive days. Female mice with two complete estrous cycles were enrolled in the experiment. Mice in the POI group were intragastrically administered with tripterygium glycosides (TGs), while those in the CON group were given an equal volume of normal saline. Samples were collected after 14 days of intervention, and GCs and cumulus‐oocyte complexes (COCs) were isolated. (B) Representative line chart of estrous cycles. (C) Rate of estrous cycle disorders, *n* = 6 mice per group. (D) Bar charts of ovarian weight comparison (left panel), *n* = 6 mice per group; and ovarian index comparison (right panel), *n* = 6 mice per group. Ovarian index (‰) = ovarian weight (mg)/body weight of mice (g) × 1000‰. (E) Comparison of serum sex hormones (AMH, E_2_, FSH, LH, T), *n* = 6 mice per group. .(F) Representative trypan blue staining image of isolated mouse ovarian granulosa cells. (G)Statistical bar chart of granulosa cell viability from three batches of COCs (COC1, COC2, COC3) via trypan blue staining.White bars stand for live cells,while black bars represent dead cells.

**Figure S4**


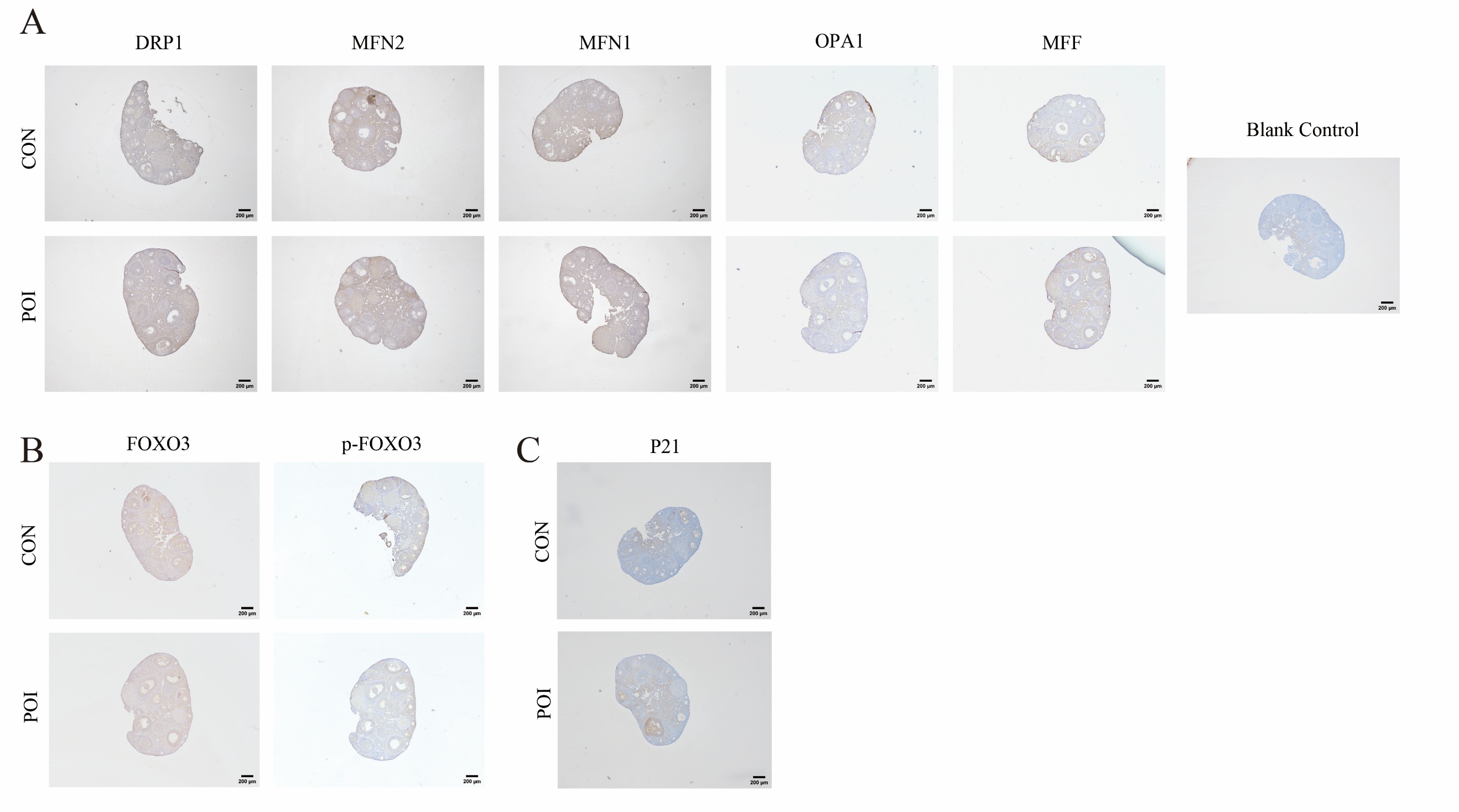


**Figure S4:**(A) Representative immunohistochemical images of mitochondrial fission‐related proteins (DRP1, MFF) and fusion‐related proteins (MFN2, MFN1, OPA1) in ovarian sections of the control (CON) and POI groups, with a blank control section shown on the right; scale bar = 200 μm. (B) Immunohistochemical staining for total FOXO3 and phosphorylated p‐FOXO3 in ovarian tissues of the CON and POI groups; scale bar = 200 μm. (C) Representative immunohistochemical images of the cellular senescence marker P21 in ovarian sections from the CON and POI groups; scale bar =200 μm.

**Figure S5**


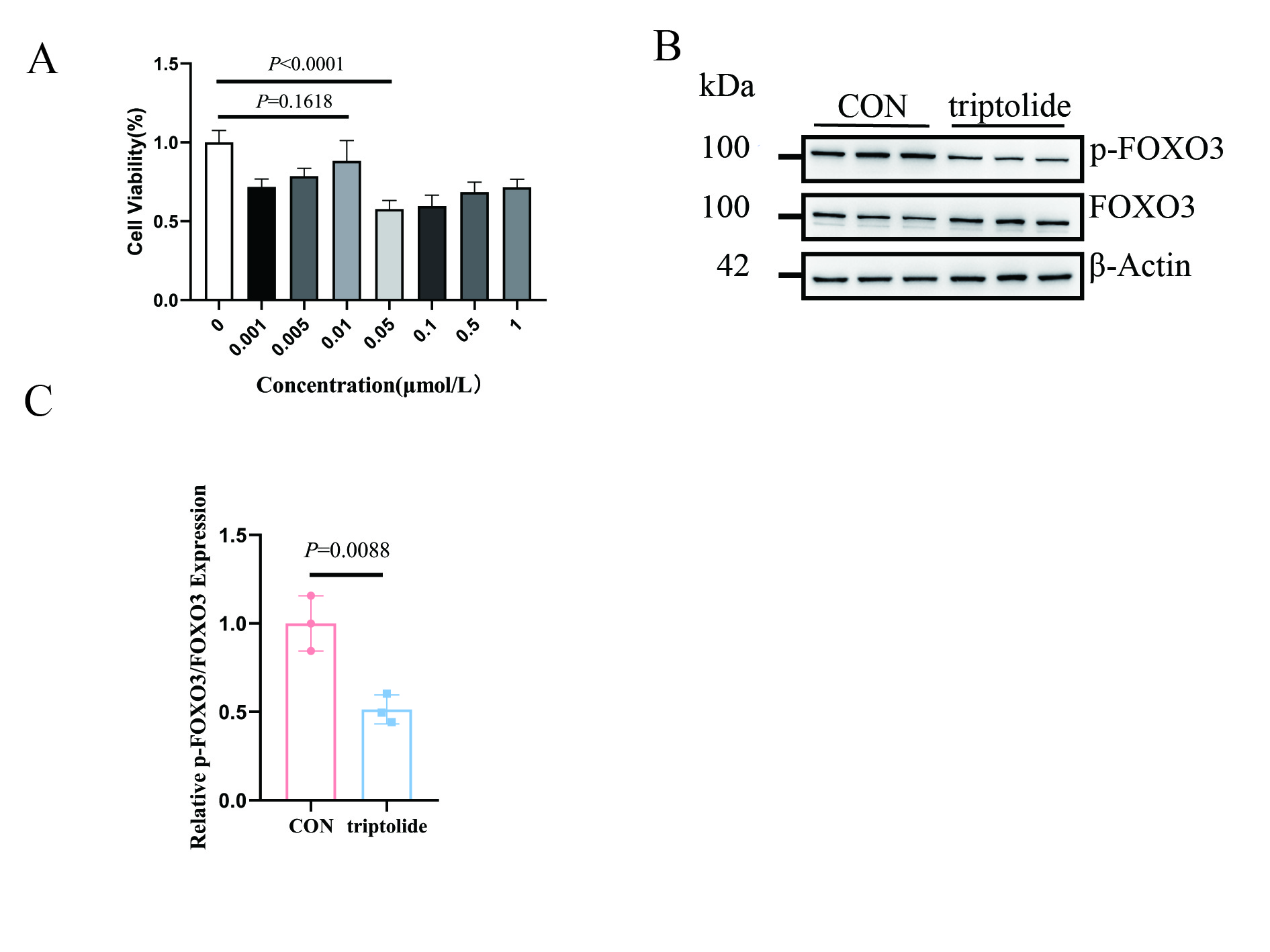


**Figure S5:**(A) Cell viability of Triptolide at different concentrations in KGN cells. (B) Representative bands of p‐FOXO3 and FOXO3. (C) Quantitative statistical analysis of ralative FOXO3 phosphorylation levels measured as p-FOXO3 normalized to total FOXO3 in CON group and triptolide group.

**Figure S6**


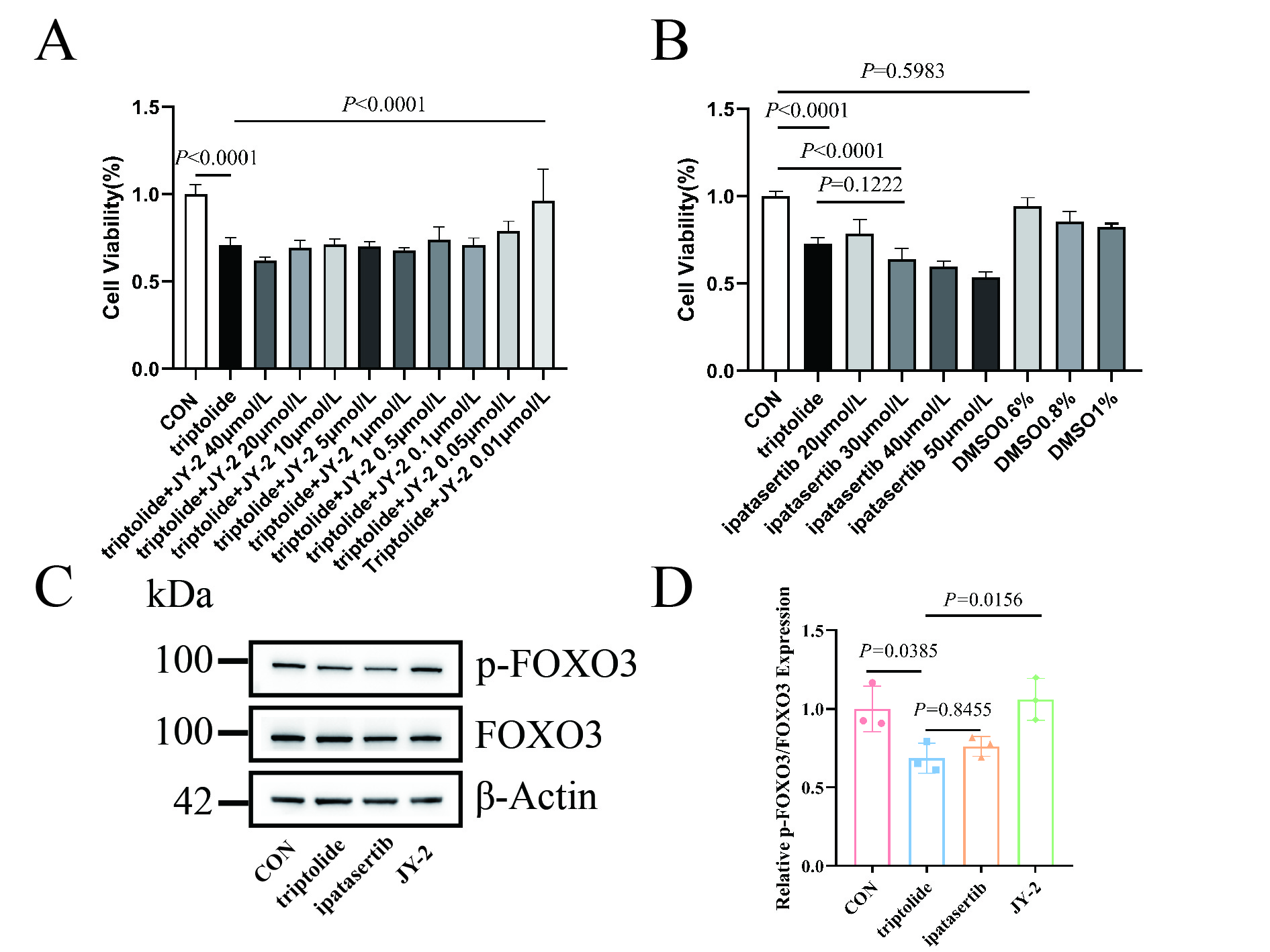


**Figure S6:**(A) Cell viability of Ipatasertib at different concentrations in KGN cells. (B) Cell viability of combined treatment with JY‐2 and Triptolide at different concentrations in KGN cells. (C) Representative bands of p‐FOXO3 and FOXO3. The lanes are arranged from left to right as follows: CON group, Triptolide group, Ipatasertib group and JY‐2 group. (D) Statistical analysis of WB gray values.

**Figure S7**


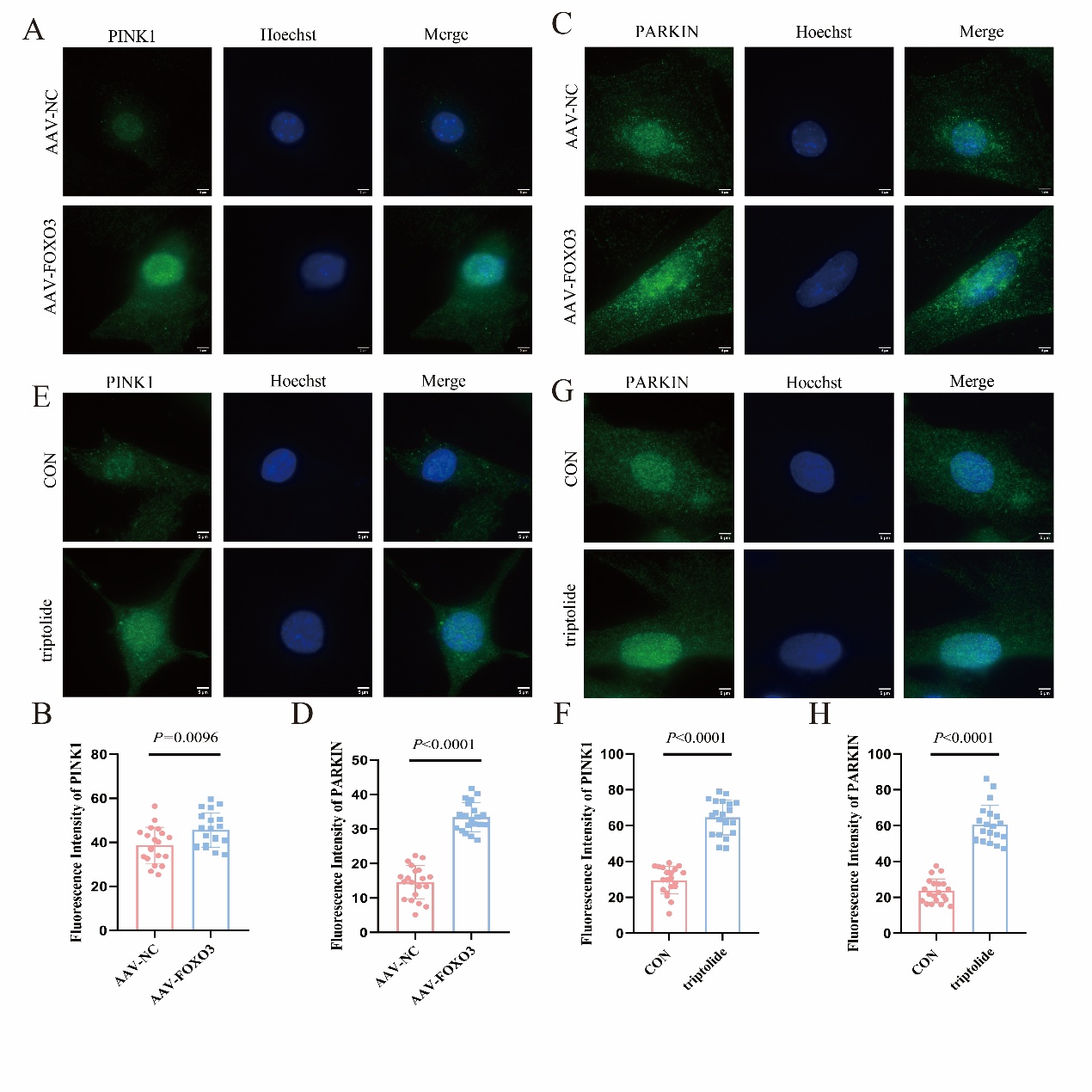
**Figure S7:** (A) Representative immunofluorescence images of PINK1 in ovarian cumulus cells from the AAV‐NC group and AAV‐FOXO3 group. Nuclei were stained with Hoechst. scale bar = 5 μm. (B) Quantitative analysis of PINK1 fluorescence intensity in the AAV‐NC group and AAV‐FOXO3 group. (C) Representative immunofluorescence images of PARKIN in ovarian cumulus cells from the AAV‐NC group and AAV‐FOXO3 group. Nuclei were stained with Hoechst. scale bar = 5 μm. (D) Quantitative analysis of PARKIN fluorescence intensity in the AAV‐NC group and AAV‐FOXO3 group. (E) Representative immunofluorescence images of PINK1 in KGN cells of the CON group and triptolide‐treated group. Nuclei were counterstained with Hoechst. scale bar = 5 μm. (F) Quantitative analysis of PINK1 fluorescence intensity in the CON group and triptolide‐treated group. (G) Representative immunofluorescence images of PARKIN in KGN cells of the CON group and triptolide‐treated group. Nuclei were counterstained with Hoechst. scale bar = 5 μm. (H) Quantitative analysis of PARKIN fluorescence intensity in the CON group and triptolide‐treated group.
